# Review of published evidence on knowledge translation capacity, practice and support among researchers and research institutions in low- and middle-income countries

**DOI:** 10.1186/s12961-019-0524-0

**Published:** 2020-02-10

**Authors:** Violet Ibukayo Murunga, Rose Ndakala Oronje, Imelda Bates, Nadia Tagoe, Justin Pulford

**Affiliations:** 1 Faculty of Health and Life Sciences, University of Liverpool, Liverpool, L69 3BX United Kingdom; 2 Liverpool School of Tropical Medicine, Center for Capacity Research, Pembroke Place, Liverpool, L35QA United Kingdom; 3 African Institute for Development Policy, 6th Floor, Block A, Westcom Point Bldg, Mahiga Mairu Ave Off Waiyaki Way, Westlands, Nairobi, Kenya; 4 KEMRI Wellcome Trust Research Programme, Kilifi, Kenya; 5 Office of Grants and Research, Kwame Nkrumah University of Science and Technology, Kumasi, Ghana

**Keywords:** Knowledge translation, Evidence, Research, Uptake, Researchers, Academic, Institution, Capacity, Evaluation, Interventions, LMIC

## Abstract

**Background:**

Knowledge translation (KT) is a dynamic and iterative process that includes synthesis, dissemination, exchange and ethically sound application of knowledge to yield beneficial outcomes for society. Effective KT requires researchers to play an active role in promoting evidence uptake. This paper presents a systematised review of evidence on low- and middle-income country (LMIC) researchers’ KT capacity, practice and interventions for enhancing their KT practice (support) with the aim of identifying gaps and informing future research and interventions.

**Methods:**

An electronic search for peer-reviewed publications focusing on LMIC researchers’ KT capacity, practice and support across all academic fields, authored in English and from the earliest records available to February 2019, was conducted using PubMed and Scopus. Selected studies were appraised using the Mixed Methods Appraisal Tool, data pertaining to publication characteristics and study design extracted, and an a priori thematic analysis of reported research findings completed.

**Results:**

The search resulted in 334 screened articles, of which 66 met the inclusion criteria. Most (*n* = 43) of the articles presented original research findings, 22 were commentaries and 1 was a structured review; 47 articles reported on researchers’ KT practice, 12 assessed the KT capacity of researchers or academic/research institutions and 9 reported on KT support for researchers. More than half (59%) of the articles focused on sub-Saharan Africa and the majority (91%) on health research. Most of the primary studies used the case study design (41%). The findings suggest that LMIC researchers rarely conduct KT and face a range of barriers at individual and institutional levels that limit their KT practice, including inadequate KT knowledge and skills, particularly for communicating research and interacting with research end-users, insufficient funding, and inadequate institutional guidelines, structures and incentives promoting KT practice. Furthermore, the evidence-base on effective interventions for enhancing LMIC researchers' KT practice is insufficient and largely of weak quality.

**Conclusions:**

More high-quality research on researchers’ KT capacity, practice and effective KT capacity strengthening interventions is needed. Study designs that extend beyond case studies and descriptive studies are recommended, including better designed evaluation studies, e.g. use of realist approaches, pragmatic trials, impact evaluations, implementation research and participatory action research.

## Introduction

Evidence-informed policy and practice can result in improved health and development outcomes, more efficient use of limited resources and greater accountability. However, decision-making is a complex process, particularly in low- and middle-income countries (LMICs), and therefore achieving the ideal of evidence-informed decision-making has been a challenge. Nevertheless, some success stories are starting to emerge, signalling a positive shift in the trend [1]. Over the past decade, several international forums have called for reforms to improve the uptake of research findings into policy and practice [2–7]. As a result, there has been increased efforts at the local, regional and international levels to bridge the ‘know–do’ gap – the difference between what is known from evidence and what is done in practice and included in policies [3]. Consequently, a specialised field concerned with promoting uptake of research into policy and practice has emerged, variously described as knowledge translation, knowledge transfer and knowledge exchange [8]. This paper adopts the term Knowledge Translation (KT) and defines it as a dynamic and iterative process that includes synthesis, dissemination, exchange and ethically sound application of knowledge to yield beneficial outcomes for society. This definition is adapted from the Canadian Institutes of Health Research [3].

It is widely acknowledged that effective KT requires researchers to play an active role in promoting evidence uptake. Various frameworks exist that conceptualise researchers’ relative role in promoting KT, including the RAPID (Research and Policy in Development programme) framework and the Framework for Assessing Country-level Efforts to Link Research to Action [9–12]. Collectively, these frameworks emphasise three overlapping and interacting dimensions that are critical in decision-making processes and that researchers can influence, namely (1) ‘political context’, relating to both the hard structures and ‘soft’ socioeconomic, political and cultural environments that shape policy processes; (2) ‘policy actors’, the key actors in a policy process, including researchers, their roles and interests as well as networks, individuals and groups who are influential in decision-making; and (3) ‘evidence’, how it is conceptualised in relation to a health issue, its credibility, methods, relevance, use, and how the message is packaged, communicated and disseminated. Specifically, researchers play a central role in producing, communicating and promoting uptake of high-quality relevant research. To achieve this, researchers are encouraged to develop and sustain relationships and regularly interact with research end-users, including policy-makers, practitioners and the public; collaborate with research end-users throughout the research process; simplify and package research findings using audience-tailored formats and platforms; and make research more easily accessible to research end-users through publishing and sharing resources in open access journals, databases and repositories [13]. For researchers to play these roles, the existence of supportive institutional and national contexts and relational processes that link and promote interaction between researchers and research end-users is critical. At national level, this relates to the extent to which researchers and other policy actors are encouraged to participate in public policy decision-making processes [12, 14–17]. At institutional level, it is the extent to which academic and research institutions prioritise KT, including having in place policies (e.g. incentives and guidelines) and budgets for KT activities as well as processes (e.g. institutional links with target audience institutions) and structures (e.g. KT units) to enable researchers to actively promote the uptake of evidence in policy and practice [10, 15, 18–20].

Several reviews have been undertaken to better understand the KT process, including the barriers and facilitators of KT, the role of context and institutions, and effective KT approaches [21–24]. However, to our knowledge, there are no reviews that have systematically synthesised the literature on researchers’ KT capacity and practice or interventions for improving their KT capacity and practice, either in general or in LMIC settings. Therefore, there is limited understanding as to the extent to which researchers are engaged in KT or what types of supports or interventions encourage and enhance their KT practice. A LMIC-specific focus is warranted given the inherent challenges related to equity that LMIC researchers and research institutions face [25–28]. For instance, health research capacity in LMICs is insufficient [25–27]. In addition, research in LMIC regions is largely funded by donors from high-income countries (HICs) and, typically, a requirement for accessing the funding is the formation of partnerships between LMIC and HIC researchers, which are led by the HIC researchers [25–28]. These issues compromise the extent to which the research produced aligns to LMIC country research priorities, is perceived as relevant and credible, and is ultimately taken up in policy and practice [27, 28].

This review paper attempts to partly address this knowledge gap by describing and synthesising published evidence on LMIC researchers’ KT capacity, practice and support. The review will contribute to a better understanding of the scope, quality and primary outcomes of the existing evidence base, thereby offering guidance to interested KT practitioners, funders, researchers and research institutions on how to strengthen KT efforts in LMIC settings.

## Methodology

We conducted a systematised review of published studies as described by Grant and Booth [29]. Our review modelled the systematic review process, except that we included all types of peer-reviewed literature without limitation to publication type and quality. This review poses the broad question – what is known about the KT capacity, practice and support among LMIC researchers and research institutions? In the remainder of this section, the steps undertaken to complete the review are outlined.

### Search strategy

A list of initial search terms was agreed upon by the authors followed by a preliminary search of the literature to test and refine the search term list. The final search terms used were ‘knowledge translation’, ‘knowledge utilisation’, ‘knowledge utilization’, ‘research uptake’, ‘research utilisation’, ‘research utilization’, ‘evidence uptake’ and ‘knowledge transfer’, combined using the Boolean term ‘AND’ with the terms ‘researchers’, ‘academics’, ‘post graduate’, ‘faculty’, ‘research centers’, ‘research organisations’, ‘research organizations’, ‘research institutions’, ‘universities’, ‘developing country’, ‘low income’, ‘low and middle income’, ‘Africa’, ‘Asia’, ‘Middle East’, ‘Latin’, ‘Caribbean’, ‘Pacific’, ‘Eastern European’ and ‘Mediterranean’. An electronic search of studies published in English was undertaken in the PubMed and Scopus databases. The search included literature from the earliest records available in the databases up to February 2019.

### Inclusion and exclusion criteria

Articles were considered eligible for inclusion if they reported studies on any or all of the following:

1. Set in LMIC settings (countries and/or academic/research institutions) as the main or one of the main settings
2. Researchers as the main or one of the main study populations, irrespective of the researcher’s nationality
3. KT capacity of LMIC researchers and/or research institutions, i.e. LMIC researchers’ interest in KT and KT expertise and skills, and institutional policies, budgets, structures and processes for carrying out KT
4. LMIC researchers’ KT practice or experience, i.e. implementation of KT activities
5. Interventions or support designed to enhance and/or facilitate the KT capacity and practice of LMIC researchers and research institutions

More than one article reporting on the same study were included if they focused on different findings. Articles reporting researchers' KT practice with study samples consisting of a mix of researchers and other professions that did not disaggregate findings by participant's profession were excluded. Articles were not excluded on the basis of publication type (commentary, review, original research).

### Screening

**Fig. 1:**
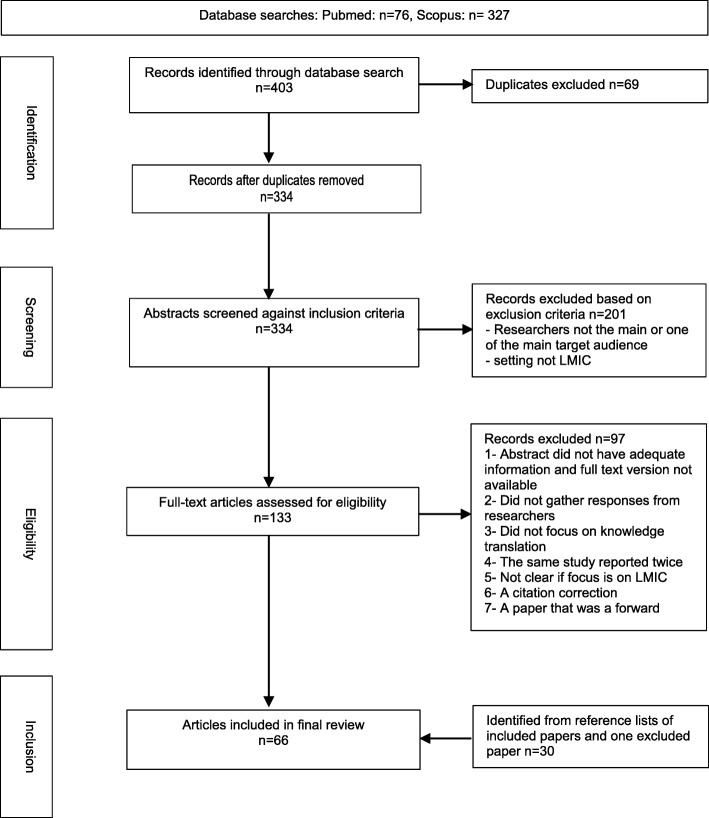
Flow chart of the identification, screening and included articles. Figure illustrates the process used to search for studies that were included in the review. Studies were searched in the PubMed and Scopus databases. ‘Identification’ shows the number of articles retrieved using the search terms (403) and after removal of duplicates (334). ‘Screening’ shows the number of articles whose titles, abstracts and keywords were screened against the study inclusion and exclusion criteria (334) and the number of articles that were excluded at this stage (201). ‘Eligibility’ shows the number of full text articles that were screened against the inclusions and exclusion criteria (133) and the number of articles that were excluded at this stage (97). ‘Inclusion’ shows that number of articles that were included in the review (66), including those that met the inclusion criteria (36) and 30 articles identified from searching the reference lists of the 36 included articles.

After removal of duplicates, the first author (VM) screened the title, abstracts and keywords of the retrieved articles against the inclusion criteria and excluded studies that were clearly not relevant. The remaining articles were read in full and screened by VM using the stated inclusion criteria. Selected articles were independently reviewed by a second author (JP). Disagreement regarding eligibility was discussed between the two authors until consensus was reached. A PRISMA (Preferred Reporting Items for Systematic Reviews and Meta-Analyses) flow chart of the identification, screening and selection process is presented below (Fig. 1).

### Quality assessment

Although studies were not excluded based on quality, we performed quality appraisal of included articles in order to aid interpretation of the strength and applicability of the evidence. The methodological quality of included primary research studies was appraised using the Mixed Methods Appraisal Tool (MMAT); the quality of commentaries and review articles was not appraised [30]. The MMAT has been designed for appraisal of qualitative, quantitative and mixed methods studies and permits concomitant appraisal of all three methodological domains. The MMAT contains two screening questions for all study designs and assesses four criteria for qualitative studies or qualitative components of mixed methods studies, four criteria for each type (randomised controlled trials, non-randomised studies and quantitative descriptive studies) of quantitative study designs or quantitative components of mixed methods studies, and three criteria for mixed methods components of mixed methods study designs. Total scores range from 25% (depicting one criterion met or none met for mixed methods studies) to 100% (all criteria met). A higher percentage score indicates a higher quality rating. The overall quality of mixed methods studies cannot exceed the quality of its weakest component. Thus, for instance, if a quantitative component of a mixed methods study scores 50% against 75% for a qualitative component, the overall score is 50%. Each study was independently rated by two reviewers (VM and NT). Discrepancies were discussed between the two reviewers (or with a third reviewer) until consensus was reached.

### Data extraction and analysis

Eligible full text articles were independently assigned by two reviewers (VM and JP) to one or more of the following three predetermined ‘topic’ categories: (1) KT capacity of LMIC researchers and research institutions, (2) LMIC researchers’ KT practice and (3) KT capacity development for LMIC researchers and research institutions. Differences in grouping the articles were discussed between the two reviewers until consensus was reached. The first author (VM) then extracted the following data using Microsoft Excel: author, year, objective, study country and region, study population, setting, study design and methods, and the main findings.

The main findings were then grouped into subthemes emerging from the literature and summarised descriptively. Tabular presentation of the data was used in some cases. Coding to determine subtheme identification and allocation was completed by the first author (VM) and verified by the second author (JP). Disagreements were discussed until a consensus was reached.

## Results

### Search results

Our searches identified 334 potentially relevant references. Following review of the titles and abstracts, we retrieved 133 articles for full text review. From these, we selected 36 articles that met the inclusion criteria. An additional 30 articles were identified through reference checks of included papers, bringing the total number of papers included for review to 66. The search process and results are illustrated in Fig. 1, the PRISMA flow chart.

### Characteristics of included studies

Of the 66 articles included in the review, 43 (65%) presented original research findings, 22 (33%) were commentaries and 1 was a structured review. A majority (*n* = 48; 73%) of the articles reported on researchers’ KT practice, 12 (18%) assessed the KT capacity of researchers or academic/research institutions and 9 (14%) reported on interventions for enhancing researchers' KT capacity and practice. More than half (*n* = 39; 59%) of the studies focused on sub-Saharan Africa as the primary study setting or as one of several study settings and a majority (*n* = 61; 93%) focused on health research.

The most common study design was some form of case study (*n* = 27; 41%). Sampling was primarily purposive (*n* = 39; 59%) and data collection was most often by in-depth interview (*n* = 23; 35%), survey (*n* = 19; 29%) or document review (*n* = 19; 29%). Data analysis was primarily thematic (*n* = 32; 48%) or descriptive (*n* = 21; 32%). An additional file presents a detailed list of included primary studies, including study design, methods, sampling, setting and population (Additional file 1).

Table 1 presents the quality assessment of the 43 original research papers according to MMAT criteria (the structured review and 22 commentaries were not assessed for quality and are not presented in the table); 39 (91%) papers had an overall quality rating of 50% or more, 12 (28%) articles received a MMAT score of 100% (indicative of ‘high’ research quality) and 4 had a score of 25% (indicative of low research quality).

**Table 1:** Quality rating of reviewed original research publications

Subcategory	No.	Overall quality rating^a^			
		25%	50%	75%	100%
KT capacity and practice	38	4	6	19	9
KT support	5	0	0	2	3
Total	43	4	6	21	12

Table illustrates the quality ratings of the 43 primary research studies included in the review. The methodological quality of included primary research studies was appraised using the MMAT with the final score expressed as a percentage. Studies can be assigned a quality rating that ranges from 25% (the lowest quality score) to 100% (the highest quality score)

*KT* knowledge translation

^a^Lowest (25%) to highest (100%) scores on MMAT scale

### Reported findings

This section presents reported findings from all 66 articles within each of the three 'topic' categories, i.e. ‘KT capacity of LMIC researchers and research institutions’, ‘LMIC researchers’ KT practice’, and ‘KT capacity development for LMIC researchers and research institutions’.

### KT capacity of researchers’ and research institutions

Twelve studies reported on the KT capacity of LMIC researchers and research institutions based on researchers’ self-reports and reviews of institutional documents [31–42]. About half of the studies were conducted in one or several countries in the Eastern Mediterranean Region [31, 33, 36, 38–40]. Three studies focused on sub-Saharan Africa (a total of six countries) [32, 34, 42] and three studies were global [35, 41] or covered more than one LMIC region, including sub-Saharan Africa and the Eastern Mediterranean Region [37]. All the studies focused on health researchers and research institutions. The capacity assessment tools and methods used varied across the studies, although a few studies used the same assessment tool [33, 36, 38, 39].

From the 12 studies reviewed, six themes emerged as follows: (1) emphasis on research production (n=9; 75%); (2) inadequate institutional links and interaction with target audience institutions (n=7; 58%); (3) inadequate capacity to communicate to non-scientific target audiences (n=6; 50%); (4) mismatch between reported and demonstrated KT competency (10; 83%); (5) influence of country income status, institutional culture, and research topic and type (6; 50%); and (6) improvement in institutional KT capacity (2; 17%).

#### Emphasis on research production

Evidence from nine studies suggests that LMIC research or academic institutions pay more attention to the research production stage of the KT process compared to the communication and dissemination stage [33, 35–42]. For example, four studies collectively assessed the KT capacity of up to 30 institutions based in nine countries in the Eastern Mediterranean Region using the same study instrument [33, 36, 38, 39]. Respondents were asked to rate three items using a five-point Likert scale (1 = low, 5 = high), namely research quality and timeliness; the existence of KT policies (e.g. incentives and guidelines), budgets, structures (e.g. department with KT expertise) and processes (e.g. links with target organisations); and researchers’ KT capacity, including training. Across the four studies, research quality and timeliness received higher mean scores, averaging 3.1/5, compared to the average mean scores for the existence of KT policies, budgets, structures and processes (1.7/5), and researchers’ KT capacity (2.4/5) [33, 36, 38, 39].

Another study by Ayah et al. [42] that assessed the institutional capacity for health policy and systems research in seven public academic institutions based in five Eastern and Central African countries found a similar trend. Respondents were asked to rate three items using a five-point Likert scale (1 = low, 5 = high), namely institutional capacity to disseminate research, institutional links with research-using institutions, and research capacity. Across the seven institutions, research capacity received higher mean scores, averaging 3.6/5, compared to capacity to disseminate research (3.1/5) and institutional links with research-using institutions (3.2/5) [42].

A similar trend was reported in a study by Lavis et al. [37] that elicited views from 308 researchers in 10 LMIC countries (China, Ghana, India, Iran, Kazakhstan, Laos, Mexico, Pakistan, Senegal and Tanzania) about their institutions’ KT support. Respondents were asked to rate their agreement, using a five-point Likert scale (1 = strongly disagree, 5 = strongly agree), about the institutional importance of KT, existence of institutional incentives supporting KT, interaction with target audiences, funding allocation for KT, existence of KT support staff and the credibility of the institution. Respondents represented four distinct research areas, including malaria prevention (*n* = 72), contraception (*n* = 94), childhood diarrhoea (*n* = 50) and tuberculosis treatment (*n* = 92) [37]. Respondents were more likely to report that their institution supports research on their research topic (*n* = 242; 81%) than researchers’ KT efforts (*n* = 205; 69%) [37].

Shroff et al. [35] assessed knowledge-generation processes in 101 institutions engaged in health policy and systems research, which are either part of the Alliance for Health Policy and Systems Research (the Alliance) network and/or were represented at the Second Global Symposium on Health Systems Research. A total of 56 countries were represented in the sample. More than three-quarters (*n* = 79; 78%) of the institutions were based in LMICs and a quarter (*n* = 25; 25%) were based in sub-Saharan Africa [35]. The assessment explored the extent to which academic incentive structures were inclusive of other formats of research products in addition to peer reviewed scientific journals. Respondents were more likely to rank publication record as the most important criteria for promotion (*n* = 44; 48%) than the ability of research to impact policy (*n* = 24; 26%) [35].

El-Jardali et al. [40] assessed the KT capacity of 223 health research institutions in 22 countries in the Eastern Mediterranean region. Respondents were asked to score the following four items using a five-point Likert scale (1 = never, 5 = always): institutional characteristics, institutional planning for research, national planning of health research, and knowledge management, translation and dissemination. A quarter of respondents reported that their institution frequently or always assesses health policy-makers’ use of their institution’s research results (*n* = 59; 27%) and the impact of their research outcomes (*n* = 52; 23%) suggesting that KT is not a strategic priority of the institutions represented in the study[40]. Gonzalez-Block and Mills [41] reported better outcomes on this. They assessed the institutional capacity for health policy and systems research in 108 research institutions based in 39 LMICs within the Alliance network. The assessment explored six strategic and interrelated groups of variables, namely institutional/country context and characteristics, institutional capacity and engagement with stakeholders, attainment of critical mass of researchers to produce quality, sustainable research, and the process of knowledge production. Three-quarters (75%) of respondents reported assessing the impact of research on policy; however, 15% reported that their efforts were unsuccessful.

#### Inadequate institutional links and interaction with target audience institutions

Evidence from seven studies (all studies described above) suggests that some LMIC research or academic institutions interact or collaborate with target audiences. However, many more institutions do not do so and, among those that do so, the focus is on a narrow range of target audiences [33, 35, 36, 39–42]. For example, in three studies [33, 36, 39] that assessed institutional KT capacity using the same assessment tool, efforts to interact with target audiences were scored in the mid to low range of the scale. Across the three studies, the average mean scores reported for various types of interaction included 2.2/5 for interaction during research priority-setting, 2.3/5 during research design and implementation, 2.3/5 during research dissemination, 2.2/5 within a network, and 2.7/5 in a government technical committee [33, 36, 39]. Ayah et al. [42] reported mixed findings in relation to formal relationships between academic institutions and target audience institutions. Across the 7 institutions that were assessed, the average mean score reported for the existence of institutional links with government policy institutions was higher (3.6/5) than that reported for links with non-government organisations (NGOs) (3.3/5), health facilities (3.1/5) and, particularly, the media (2.5/5). In addition, the average mean score reported for individual interaction and communication with decision-makers/policy-makers was 3.2/5 [42].

Similarly, El-Jardali et al. [40] found that less than half of respondents reported that their institution frequently or always involves policy-makers and stakeholders when setting priorities for research on health (*n* = 79; 41%) and translates high priority policy concerns into priority research questions (*n* = 84; 43%). In addition, around a third (*n* = 67; 34%) of respondents reported that their institution involves policy-makers and stakeholders in research projects, including in the development of joint proposals, study design and data collection tools, analysis and writing up of publications [40].

Likewise, Shroff et al. [35] assessed the existence of formal (memoranda of understanding or commissioned research) or informal (personal interactions) linkages. A majority (*n* = 94; 93%) of respondents reported the existence of either formal or informal linkages with National or State level Ministries of Health or public health bodies that aimed to produce research to inform policy design and implementation [35]. However, less than half (*n* = 46; 46%) of the respondents reported the existence of formal linkages that bring researchers and decision-makers together to identify relevant research areas [35].

Gonzalez-Block and Mills [41] also explored engagement with stakeholders and found that a majority (70%) of respondents reported interaction using external boards or advisory bodies [41]. However, the types of groups involved in these platforms was narrow. Out of 11 key groups that were explored, respondents mainly reported the involvement of health authorities and their staff (35%) and government, international experts and other government bodies (25%). Financing agencies (8%) and NGOs (7%) were among the least engaged groups. Furthermore, 95% of respondents reported continuously communicating with stakeholders, involving stakeholders in research and securing the presence of researchers in key health policy debates. However, they reported having lower capacity to implement these activities compared to simply raising awareness of research results and recommendations among stakeholders.

#### Inadequate capacity to communicate to non-scientific target audiences

Evidence from six studies (all described above) suggests that the capacity of LMIC research institutions to tailor and communicate research findings to non-scientific target audiences is inadequate and/or focuses on a narrow range of target audiences [33, 34, 36, 39, 40, 42]. For example, in three studies [33, 36, 39] that assessed institutional KT capacity using the same assessment tool, skills for communicating research and use of accessible communication formats were scored in the mid to low range of the scale. Across the three studies, the average mean scores were 2.4/5 for the existence of research communication skills among researchers, 2.6/5 for the extent to which researchers convert research findings into actionable messages appropriate to the target audience, 2.3/5 for the extent to which websites or electronic databases are used to make research available, and 2/5 for the extent of regular communication with media and target audiences through non-scientific publications [33, 36, 39].

El-Jardali et al. [40] reported a similarly low capacity in research institutions to communicate findings to non-scientific target audiences. The study's respondents were more likely to report that their institution frequently or always disseminates research through traditional academic platforms, including publishing in their institutions’ (*n* = 99; 44.4%) or other (*n* = 131; 59%) peer-reviewed journals, seminars or conferences (*n* = 143; 64%), their institutional websites (*n* = 118; 53%) and newsletter, email or printed reports circulated within the institution (*n* = 98; 44%). Other platforms more accessible to non-scientific target audiences were more likely reported as never or rarely used to disseminate research findings to policy-makers and other stakeholders, including letters/briefs/tailored messages (*n* = 89; 40%), policy briefs (*n* = 108; 48%), and policy dialogues (*n* = 108; 48%). Likewise, study respondents were more likely to report that their institution frequently or always communicates research findings to other researchers (*n* = 89; 39.9%), policy-makers in government (*n* = 81; 36%), and healthcare providers such as clinicians, nurses and pharmacists (*n* = 81; 36%) [40]. In turn, study respondents were more likely to report that their institution never or rarely communicates research findings to directors of donor agencies (*n* = 134; 64%), international agencies (*n* = 123; 55%), NGOs (*n* = 120; 54%) and the public (*n* = 98; 44%) [40].

Similar trends were reported by Ayah et al. [42] and Simba et al. [34]. Across the seven institutions assessed in the two studies, the average mean score reported for the existence of a strong communications staff with capacity to effectively communicate research findings to many target audiences fell in the middle range of the scale (2.8/5) [42]. However, an audit of publications produced by the institutions revealed that publication outputs were mainly peer-reviewed journal articles [34]. Production of tailored communication outputs, such as policy briefs, reports to government agencies, press releases, media briefs and multimedia products, was negligent [34].

#### Mismatch between reported and demonstrated KT competency

Evidence from ten studies [31–34, 36, 37, 39–42] suggests that the KT skills of LMIC researchers are often inadequate. Across three studies [33, 36, 39] that used the same assessment tool (studies described above), the average mean scores reported for the existence of KT training needs assessment (2.1/5) and KT training within research methods training programmes (2/5) fell in the low range of the scale [33, 36, 39]. Nevertheless, familiarity of KT concepts and what it entails was scored in the middle range of the scale (2.6/5) suggesting likely over-estimation of researchers' KT capacity among respondents [33, 36, 39]. El-Jardali et al. [40] also assessed the dissemination skills of researchers in the institutions. At least two-thirds of respondents reported the existence of skills to disseminate research findings to policy-makers (*n* = 140; 63%) and directors of NGOs (*n* = 167; 75%) [40]. However, similar to other studies, these findings contradicted the actual communication and dissemination activities and formats reported suggesting an over estimation of researchers' KT capacity among respondents [40].

Ayah et al. [42] and Simba et al. [34] (study described above) reported lower average mean scores for the availability of time to disseminate research findings (3.2/5) and the motivation (3.1/5) and skills (3.2/5) to do so than those reported for research capacity (3.6/5) [42]. However, similar to other studies, researchers’ competency in KT was scored higher in relation to the actual products and activities researchers are engaging in [34]. This further supports the likely overestimation of researchers’ KT capacity.

Gonzalez-Block and Mills [41] (study described above) reported some efforts to enhance researchers’ KT capacity. Most respondents reported that their institution had implemented capacity development strategies aimed at improving researchers’ awareness of policy issues and processes (95%) and ensuring the production of health policy and systems research dissemination materials (85%). However, similar to other studies, the study found gaps in KT competence among researchers, particularly in relation to interaction with a broader range of target audiences.

Similarly, inadequate capacity for undertaking systematic reviews was reported in a study by Yousefi-Nooraie et al. [31]. The study assessed the views of 131 Iranian clinical and healthcare researchers, health policy and decision-makers, research policy-makers (directors and managers) and support staff on how the development and usage of evidence from systematic reviews can be promoted in a country with limited resources. The article did not report the sample size by profession. Respondents were asked to select five important items from a list of 20 and suggest interventions to address them. ‘Competency of researchers to conduct systematic reviews’ was one of four top ranked issues needing to be improved.

Two studies suggest that despite having inadequate KT knowledge and skills, researchers understand the importance of KT and are supportive of their role in promoting KT. Lavis et al. [37] (study described above) assessed researchers’ attitudes towards KT practice. Respondents agreed or strongly agreed that KT activities should be done collaboratively with target audiences (71%), suggesting a positive attitude to collaboration with target audiences and an understanding of its value [37]. Similarly, Uneke et al. [32] assessed the KT practice of six Nigerian senior academic researchers. Respondents were asked to score their preparedness to partner with policy-makers in the policy-making process using a four-point Likert scale and scored it high (3.8/4), suggesting that they hold a positive attitude towards collaboration with target audiences and an understanding of its value.

#### Influence of country income status, institutional culture, and research topic and type

Evidence from six studies (all studies described above) suggests an influence of several factors that facilitate or hinder KT practice, including country income status [36, 40], institutional culture [35, 41, 42], research topic and research type [37]. Maleki et al. [36] found that institutions based in low-income countries had lower mean scores across all KT items assessed compared to those based in middle- income countries. El-Jardali et al. [40] found that respondents from institutions based in upper middle-income countries were more likely to report a lack of national research priorities, whereas those from institutions based in LMICs were more likely to report not knowing whether their countries had national health research priorities. These findings suggest less KT capacity among academic and research institutions in low-income countries compared to those in higher-income countries.

In the study by Ayah et al. [42], the institution with the lowest scores was based in the same country as one of the three higher scoring institutions, suggesting an influence of institutional culture. In addition, country context emerges as a likely influencing factor in studies that assessed the institutional KT capacity of health research institutions based in different countries using the same assessment tool but recording varying levels of capacity [33, 36, 38, 39]. Studies by Shroff et al. [35] and Gonzalez-Block and Mills [41] also tended to report more positive findings in relation to other studies reviewed in this section, particularly in relation to incentives [35], capacity development efforts and efforts by institutions to assess the impact of KT efforts [41]. In both studies, the study populations were part of the Alliance for Health Policy and Systems Research network, suggesting an influence related to being a part of the network. Finally, Lavis et al. [37] found that respondents undertaking childhood diarrhoea research consistently fared worse than other researchers on most of the KT capacity items that the study assessed and yet were also more likely to believe that their research was ready for application. This suggests an influence of the type of research being undertaken and the related research culture.

#### Improvement in institutional KT capacity

Evidence from a few studies [35, 37] suggests that KT prioritisation in research institutions is improving over time. Lavis et al. [37] found that as many respondents reported a positive shift over time in their institution’s support for the research being undertaken (*n* = 202; 68%) as those that reported a positive shift over time in their institution’s support for researchers’ KT efforts (*n* = 188; 64%). Shroff et al. [35] found that a third (*n* = 36; 36%) of respondents reported the existence of incentives in their institutions encouraging researchers to carry out policy-relevant research. In addition, two institutions reported the creation of alternative career tracks for policy-relevant research with career advancement not linked to publication in high-impact journals. Furthermore, around a third (*n* = 30; 30%) of respondents reported being required to convert research findings into recommendations for policy-makers [35].

### LMIC researchers’ KT practice

Factors influencing researchers’ KT practice were reported in 47 articles. From the 47 papers reviewed, five themes emerged, namely (1) KT activities undertaken by LMIC researchers (*n* = 6; 13%); (2) factors influencing LMIC researchers’ KT practice (*n* = 40; 85%); (3) barriers to LMIC researchers’ KT practice (*n* = 39; 83%); (4) facilitators of LMIC researchers’ KT practice (*n* = 38; 81%); and (5) strategies recommended for improving LMIC researchers’ KT practice (*n* = 38; 81%).

#### KT activities undertaken by LMIC researchers

Six studies used various assessment tools and methods to quantify LMIC researchers’ KT activities [32, 37, 43–46]. Two studies were global studies involving a mix of HIC and LMIC regions and countries [46] and one study focused on 10 LMICs [37]. The remaining studies were based in one LMIC each located in Africa [32], Asia [43, 45] and the Eastern Mediterranean region [44]. All except one study focused on health research [45]. The findings suggest that researchers are undertaking a narrow range of KT activities and, in particular, investing little time to interact with and tailor and target their findings to different target audiences. Across the studies, the most frequently reported dissemination formats were scientific publications and conferences. Media was the least reported dissemination avenue. In addition, the studies reveal that the type of research influences the extent to which researchers are involved in KT activities.

For example, Nedjat et al. [44] assessed the frequency of ‘passive’ and ‘active’ KT activities implemented by 208 researchers involved in basic (*n* = 46; 22%), clinical (*n* = 101; 49%) and health systems (*n* = 61; 29%) research at the Tehran University of Medical Sciences in Iran. Respondents were more likely to report undertaking passive KT activities than active KT activities. Among the ‘passive’ activities assessed, most respondents reported publishing articles in domestic (*n* = 130; 63%) and international (*n* = 101; 49%) peer-reviewed journals and presenting findings in conferences, seminars and domestic meetings (*n* = 100; 48%). Among the ‘passive’ KT activities targeting non-scientific audiences assessed, sending either the full or summaries of research reports to users was the most commonly reported approach used by respondents (*n* = 93; 45%). Respondents were least likely to report displaying their research results on a website (*n* = 39; 19%) or mailing or e-mailing articles or reports or summaries to stakeholders (*n* = 15; 7%) and/or publishing research results in newspapers (*n* = 8; 4%). Among the active KT activities assessed, only 15% (*n* = 32) of respondents reported producing user-friendly products such as plain writings for patients, special texts for managers, practical reports for clinical and lab colleagues, or special reports for industrial managers or academics; 10% or less of respondents reported presenting results to media, including reporters, radio and TV (*n* = 16; 8%) and holding briefings with stakeholders for the presentation of research results (*n* = 21; 10%). Health systems researchers were more likely than basic and clinical researchers to report undertaking passive and active KT activities targeting non-scientific audiences. This suggests that the type of research a researcher is involved in influences the extent of their KT practice.

Similarly, Lavis et al. [37] found that researchers were engaging more in KT activities that were less interactive and requiring less effort. They assessed the KT activities of 308 researchers in 10 LMICs (China, Ghana, India, Iran, Kazakhstan, Laos, Mexico, Pakistan, Senegal and Tanzania). The study explored three broad categories of KT activities, namely (1) ‘producer push’ efforts (what is communicated to target audiences outside the research community, to whom, by whom, how and with what effect); (2) efforts to facilitate ‘user pull’ (strategies used to provide access to research and to develop target audiences’ capacity to use research); and [3] exchange efforts (target audience involvement in research and KT activities). Half (*n* = 161; 52%) of the respondents reported that they actively conduct KT activities. The study found that respondents more frequently undertook ‘producer push’ KT activities than activities that facilitate ‘user pull’ or exchange activities. Respondents reported undertaking the following KT activities most frequently: developing research products that used language appropriate to specific target audiences (*n* = 167; 57%); messages specifying specific actions (*n* = 174; 57%); reviewing information that described the needs or goals of specific target audiences (*n* = 165; 55%); involving target audiences in the research process, including implementation (*n* = 182; 60%), analysis (*n* = 170; 56%), development of research products (*n* = 177, 59%) and in KT activities (*n* = 173; 57%); and attending conferences and workshops (*n* = 168; 55%) and events organised by target audiences (*n* = 162; 54%). Less than half of respondents reported undertaking interactive activities outside the research process such as participation in government-sponsored meetings (*n* = 123; 41%) and expert committees or groups (*n* = 126; 42%). The least frequently reported KT activities included publishing research on non-scholarly publications (*n* = 68; 23%), accepting requests from journalists to participate in interviews or debates (*n* = 73; 25%), mailing or emailing research to target audiences (27% or less), making research available on a website (19% or less), providing systematic reviews of research literature (*n* = 79; 27%), and maintenance of some reserve capacity (financial or human resources) to conduct short-term research projects in response to target audience requests (*n* = 58; 20%) [37].

Little interaction between researchers and target audiences, specifically policy-makers, was also reported by Uneke et al. [32], who assessed the KT practice of six Nigerian senior academic researchers. Respondents were asked to rate several KT practice items using a 4-point Likert scale (1 = low, 4 = high). Only one item, the relevance of research to health policy-making in Nigeria, received a perfect score of 4/4. The rest of the items received scores of 2 or less out of 4, including the existence of a partnership between researchers and health policy-makers (1.5/4), the frequency of previous research findings being made available to policy-makers (1.67/4) and the frequency of being consulted by policy-makers for research evidence (2/4). In addition, only one of the six researchers reported having experience participating in policy-making processes [32].

Similarly, a study by Lashari et al. [45] assessed the KT efforts of 24 PhD faculty members from eight Indian universities offering degrees in the fields of environmental engineering and environmental sciences. Respondents were asked to score the frequency of several KT activities using a scale of 1 (never) to 3 (at most), including promoting KT through publications, networking, mobility of researchers, joint research projects, intellectual property, co-operations, and infrastructure of the university. Survey respondents scored disseminating their research via peer-reviewed scientific publications (2.9/3) higher than through professional publications (2.1/3) [45]. The article did not define what professional publications refer to. However, elsewhere [47], they are described as articles written for specific audiences such as managers or administrators in business, finance and industry, often published on a weekly or monthly basis. Respondents also scored their interaction with industry staff in conferences and workshops (2.3/3) higher than having personal (informal) contacts with industry (2.1/3) [45]. They also scored their co-operation with other universities or higher education institutions (2.4/3) and other departments within their university (2.6/3) higher than with commercial laboratories or enterprises (1.7/3) and commercial manufactures or service providers (1.4/3). Respondents also scored their university sharing facilities with industry (2.3/3) higher than their university’s technology/knowledge transfer office organising KT activities (1.5/3) and establishing spin-offs (1.2/3).

Likewise, Walugembe et al. [43] explored the KT activities of 13 reproductive health researchers’ based at the International Centre for Diarrhoeal Disease Research (ICDDR) in Bangladesh. A majority (*n* = 12; 92%) of respondents reported using dissemination workshops to share their evidence and half (*n* = 7; 53%) reported publishing scientific papers. All respondents reported undertaking other activities to package their findings and to ensure that their key findings were made accessible to stakeholders. These included the production of fact sheets, sharing findings on the website, engaging service providers, joining advocacy networks, and producing wind banners, among others. However, fewer respondents reported developing policy briefs (*n* = 6; 46%), having one-on-one meetings with policy-makers (*n* = 3; 23%), providing technical assistance to policy-makers (n = 3; 23%), producing research reports (n = 3; 23%) and engaging the media (*n* = 2; 15%). The study also found that few of the respondents reported knowing how and at what stages of the policy-making process their findings were utilised, suggesting that they do not systematically assess the impact of their KT activities.

Cheung et al. [46] conducted a print media analysis in the 44 countries in Africa, the Americas, Asia, and the Eastern Mediterranean that host (or have signalled their intent to host) a local EVIP-Net or similar type of KT platform. The analysis aimed to assess whether and how policy-makers, stakeholders and researchers talk in the media about three topics, namely policy priorities in the health sector, health research evidence and policy dialogues regarding health issues. The assessment identified 5.5 and 5 times more articles describing health research evidence (1468) than those describing government policy priorities (264) and policy dialogues (290), respectively. Of the 264 articles mentioning policy priorities, researchers were least likely to mention government policy priorities (*n* = 6; 2%) compared to government officials (*n* = 208; 79%) and stakeholders (*n* = 27; 10%). This suggests that it is uncommon for researchers to ensure that their research aligns to government policy priorities. Of the 1468 articles describing health research evidence, 569 (39%) described the type of study. Systematic reviews were the least mentioned study type (*n* = 31; 5%) compared to basic science (*n* = 226; 40%), observational studies (*n* = 185; 33%) and randomised control trials (*n* = 115; 20%). This suggests that not much effort is going into synthesising the evidence base on issues to inform policy and practice decisions, processes that should ideally be based on this kind of evidence. Of the 290 articles describing policy dialogues addressing issues in the health sector, researcher involvement in the dialogues was least mentioned (*n* = 27; 9%) compared to involvement of government officials (*n* = 287; 99%) and stakeholders (*n* = 283; 98%). This suggests that active interaction between researchers and policy actors is uncommon.

#### Factors influencing LMIC researchers’ KT practice

Table 2 presents the factors influencing LMIC researchers’ KT practice most commonly cited across 40 papers included in this subtheme. As shown, a few papers illustrate successful examples of LMIC researchers getting their research used by target audiences [43, 48, 49]. Most papers that reported on the extent of the use of evidence by target audiences stated that it is an uncommon practice. Researchers’ interest in KT and institutional incentives promoting KT were the most commonly cited factors influencing LMIC researchers' KT practice. Equally, the credibility of researchers, their institution and the research they produce, as perceived by target audiences, was cited as critical.

**Table 2:** Reported factors that influence knowledge translation (KT) as reported by low- and middle-income country (LMIC) researchers

	Papers citing issue
Research use	
Research not always used to inform policy and practice decisions	[48–56]
Examples of LMIC researchers successfully getting evidence used	[43, 48, 49]
Factors influencing KT	
Researchers’ reputation/credibility/contextual understanding	[32, 49, 50, 57–68, 127]
Relevance and credibility of research evidence	[32, 49, 50, 53, 54, 58, 60, 62–67, 70–72, 127]
Contrasting views, demands and incentives among researchers and policy-makers in relation to research, its use, policy actor roles and policy-making	[32, 49, 50, 57, 59–61, 67, 70, 73–78]
Nature of policy issues (technical versus contested versus interest of policy-maker)	[49, 58–62, 67, 70, 76, 79]
Political context	[32, 48–51, 53, 58, 59, 62, 64, 67, 80, 81, 127]
Decision-makers’ research background	[51, 55, 57, 65, 77]
Donor influence	[51, 54, 55, 57–60, 70, 71, 127]
International influence, e.g. WHO	[54, 58, 59, 65, 67, 70, 71, 75, 77, 80, 82–84]

Table illustrates extent of use of research and factors influencing use of research that were commonly cited by researchers in the studies included in the review. A listed factor was considered commonly cited if it was reported in three or more studies

#### Barriers to LMIC researchers’ KT practice

Table 3 presents the barriers to LMIC researchers’ KT practice most commonly cited across the 39 papers included in this subtheme. As shown, limited funding for production of policy-relevant research and undertaking KT activities and researchers’ inadequate KT capacity were the most frequently mentioned barriers to KT practice among LMIC researchers.

**Table 3:** Reported barriers of knowledge translation (KT) as reported by low- and middle-income country LMIC) researchers

Barriers	Papers citing issue
Political context	
Short window for responding to policy demands	[49, 50, 51, 57, 59, 67, 70, 77, 78, 80]
High turnover of government officials and politicians	[49, 50, 53, 59, 62, 64, 67, 81, 85, 127]
Unfavourable political environment	[49, 50, 53, 58, 60–62, 64, 67, 68, 70, 74, 80, 81, 127]
Policy implementation challenges	[49, 50, 53, 58, 64, 78, 80, 81]
KT knowledge and skills of target audiences	
Target audiences lack knowledge/understanding and skills related to research and policy development	[31, 49, 50–52, 55, 57–59, 64, 67, 68, 78, 82, 84, 86]
Research availability, accessibility and relevance	
Limited access to and/or inadequate relevant evidence	[31, 48, 50–52, 58, 60, 61, 70, 77, 78, 87, 88, 127]
Researcher/target audience collaboration and networking	
Inadequate interaction between researchers and policy-makers	[37, 49–52, 74, 77, 88]
Researchers’ KT knowledge, attitudes and skills	
Researchers’ inadequate research and KT skills	[31, 49, 50, 52, 55, 61, 67, 72, 78, 87, 127]
Researchers’ fear of, or limited engagement of, media	[52, 67, 74, 77]
Challenges with simplifying research findings	[49, 50, 55, 59, 60, 64, 67, 71, 74]
Researcher not interested in KT	[48, 49, 59–61]
Research institutional support	
Inadequate institutional support and incentives for KT	[31, 32, 43, 44, 49–51, 63, 67, 74, 78]
Funding	
Limited funding for production of relevant research and KT activities	[31, 32, 37, 44, 49–52, 56, 57, 59, 60, 63, 67, 78, 80, 81, 89]

Table illustrates the barriers to the use of research findings commonly cited by researchers in the studies included in the review. A listed barrier was considered commonly cited if it was reported in three or more studies

*KT* knowledge translation

#### Facilitators of LMIC researchers’ KT practice

Table 4 presents the facilitators of LMIC researchers’ KT practice most commonly cited across the 38 papers included in this subtheme. Collaborating and networking with target audiences was the most frequently cited facilitator of LMIC researchers’ KT practice. How researchers communicate their research, i.e. whether it is tailored and targeted for different audiences and provided at opportune times, was the second most cited facilitator of LMIC researchers' KT practice.

**Table 4:** Reported facilitators of knowledge translation (KT) as reported by low- and middle-income country (LMIC) researchers

Facilitators	Papers citing issue
Political context	
Political and institutional requirements to the use of evidence	[49–51, 57, 58, 61, 64, 65, 72]
Favourable political environment	[49–51, 57–59, 64, 68, 80, 84]
KT knowledge and skills of target audiences	
Training/sensitisation of target audiences, e.g. policy-makers, communities, media etc.	[56–58, 60, 62, 64, 74, 78, 81, 82]
Technical support to policy-makers and implementers	[43, 48, 78, 81]
Research availability, accessibility and relevance	
Timely research	[48, 49, 58, 61, 64, 65, 71, 72, 85, 87, 127]
Availability of policy-relevant research	[43, 50, 57, 64–66, 71–73, 77, 80–82, 87]
Researcher/target audience collaboration and networking	
Researchers collaborating with policy-makers and other stakeholders	[49–51, 53, 54, 56–64, 66–68, 71, 73–75, 77, 78, 80–82, 84, 87, 89, 127]
Researchers interacting with target audiences through existing networks or strategic alliances and championing issues	[43, 49–53, 57–62, 64, 65, 67, 70–72, 74, 75, 77, 78, 80, 82, 84, 85, 127]
Researcher interacting with research users through informal networks and personal relationships	[37, 49, 50, 58, 59, 62, 71, 72, 74, 85]
Trust between policy-makers and researchers	[49, 50, 62, 66, 67, 71, 73, 74, 82]
Target audiences involved at various stages of the research process	[43, 48, 50, 53, 56–58, 63, 64, 66–68, 70, 71, 73, 78, 81, 82, 85]
Researchers involved in policy formulation and implementation	[58, 61, 67, 70, 72, 84]
Researchers placed in key decision-making positions in government or in close proximity with national programmes	[59, 67, 70, 75]
Research communication	
Targeted dissemination of research findings	[49, 51–53, 56–58, 62, 63, 65, 68, 71, 74, 77, 80–82]
Tailored messages for various audiences	[50, 53, 57, 58, 63, 64, 67, 70–72, 74, 75, 77, 81, 82, 127]
Framing of research findings in context	[50, 53, 57, 58, 64, 67, 71, 77, 81, 82, 127]
Media engagement activities, e.g. newspaper articles, TV and radio shows	[43, 50, 62, 67, 82]
Use of credible messengers/knowledge intermediary	[48, 49, 50, 57, 58, 70, 71, 77, 84, 85]
Use of policy windows	[48, 50, 58, 67]
Funding	
Funding available for research and KT	[50, 53, 56, 58, 59, 61, 62, 64, 66, 78]

Table illustrates the facilitators of use of research findings commonly cited by researchers in the studies included in the review. A listed facilitator was considered commonly cited if it was reported in three or more studies

#### Strategies recommended or used to improve LMIC researchers’ KT practice

Table 5 presents the strategies used or recommended to improve LMIC researchers’ KT practice commonly cited across the 38 papers included in this subtheme. The most cited strategies for improving LMIC researchers' KT practice were collaboration and networking between researchers and target audiences, tailored and targeted communication of research, strengthening of researchers’ KT capacity through training, sensitisation and partnership, and availability or allocation of more funding for the production of relevant research and KT activities.

**Table 5:** Recommended knowledge translation (KT) strategies as reported by low- and middle-income country (LMIC) researchers

Strategies	Papers citing issue
KT knowledge and skills of target audiences	
Sensitise and train target audiences on the value of research use in decision-making and research methods	[31, 50, 51, 56, 57, 62, 64, 67, 70, 74, 75, 78, 84, 85]
Research availability, accessibility and relevance	
Rapid response service, strategic consultancy and research commissions for policy institutions and donors/funders	[49, 53, 57, 59, 61, 64, 67, 77, 87, 127]
Produce a mix of research evidence, including operations research, systematic reviews, effectiveness and cost-effectiveness research, develop low cost innovations to improve practice, etc.	[49, 53, 57–59, 61, 64, 67, 70–72, 74, 75, 77, 80, 81, 85]
Researcher/target audience collaboration and networking	
Establish strong links with institutions involved in the decision-making process in research	[49, 57, 58, 60, 61, 64, 67, 72, 74, 75, 85, 88]
Involve target audiences in the research process	[53, 56, 58, 61, 62, 64, 72, 75, 77, 85, 89]
Establish KT platforms or participatory workshops for discussing research findings	[31, 53, 57, 75, 78, 88]
Research communication	
Tailor and target the dissemination of research	[53, 55, 57, 58, 61–63, 71, 74, 77, 80, 86]
Stakeholder analysis/analysis of policy-making process	[49, 53, 61, 64, 67, 72, 80]
Identify and seize windows of opportunity	[53, 58, 62, 64, 67, 71, 75]
Develop communication strategy	[72, 82, 89]
Researchers’ KT knowledge, attitudes and skills	
Strengthen research and KT capacity	[31, 49, 50, 57, 58, 67, 74, 85, 88, 89]
Forge strategic partnerships with international research institutions	[31, 70, 78, 84]
Forge strategic partnerships with influential people, media and knowledge brokers	[54, 57, 61, 67, 74, 80, 88, 89]
Research institutional support	
Strengthening institutional support and incentives for KT	[57, 72, 74, 77, 78]
Funding	
More funding allocated to KT and production of policy-relevant research	[31, 44, 49–52, 56–58, 62–64, 67, 68, 74, 75, 78, 80, 89, 127]

Table illustrates the recommended strategies for improving the use of research findings commonly cited by researchers in the studies included in the review. A listed recommendation was considered commonly cited if it was reported in three or more studies

### KT capacity development for LMIC researchers and research institutions

Nine studies described and/or evaluated interventions or tools aimed at enhancing LMIC researchers’ KT practice [69, 72, 90–96]. A summary of each study is presented in Table 6. Six studies reported either the formation or evaluation of KT interventions, which varied considerably in terms of the interventions used, target populations, length and outcome measurements reported, as described below [69, 90–94]. Two studies reported on the same intervention but focused on different aims, assessment/evaluation duration and outcomes measured (feasibility assessment versus process evaluation) [91, 92]. Three studies presented tools for enhancing KT practice and recommended their application and evaluation by researchers and research institutions [72, 95, 96]. Collectively, the interventions/tools focus on enhancing KT at various levels, including the systems level [90, 94], institutional level [72, 91, 92], individual level [93] and activity level [69, 95, 96]. Systems level interventions represent government-led interventions with substantial involvement of academic or research institutions. Institutional level interventions represent those initiated and implemented by academic or research institutions. Individual level interventions aim to improve individual KT knowledge and skills. Activity level interventions are guidelines for implementing specific KT activities such as development of policy briefs, organising policy dialogues and pairing researchers with policy-makers to enhance their interaction.

**Table 6:** Summary of papers describing/evaluating knowledge translation (KT) interventions/tools

Intervention level/paper	Intervention and evaluation aims, design and results/recommendations
Systems level	
Majdzadeh 2010 [94]	- Intervention: In 1985, the Iranian government integrated medical schools into the Ministry of Health resulting in the creation of the Ministry of Health and Medical Education (MOHME) - Aim: To enhance translation of evidence into policy and practice - Evaluation: Qualitative study involving interviews and focus group discussions with decision-makers, non-medical professionals, researchers (from intervention and control settings) and practitioners to assess impact on MOHME decision-making processes - Results: Increased operations research but institutional policy-making culture remained unchanged; time for teaching and research compromised because of over-emphasis on service delivery - Recommendations: Need for establishment of clear regulations and incentives to guide and promote the integration
Sriram 2018 [90]	- Intervention: Processes to form National Knowledge Platform (NKP) initiated in 2013, in India, by the Ministry of Health and Family Welfare (MOHFW) - Aim: To enhance dialogue and exchange between policy-makers and health policy and systems researchers to inform the generation of policy-relevant evidence and decision-making - Evaluation: Qualitative case study involving interviews with researchers and policy-makers and document reviews in 2016 to analyse the policy-making process - Results: NKP initially established as embedded within MOHFW but evolved into an independent platform; researchers network at the forefront, pushing formation of NKP, including leading proposal development; researcher network initially considered as secretariat but later involved as Steering Committee member
Institution level	
Mijumbi 2014 [91]	- Intervention: University-based (Makerere University) rapid response service (RSS), implemented from 2010 to present, targeting state and non-state decision-makers, including mid- and top-level officials at Ministries of Health, civil society organisations and legislators - Aim: Timely (within 28 days) development of four-page evidence briefs with clear key messages to support health systems policy development and planning - Feasibility assessment: Case study involving RSS service data and interviews with service users covering the period 28 months from start of the service - Results: Among nearly half of the policy-makers, the intervention resulted in a change in the course of action based on the evidence provided in the rapid response briefs
Mijumbi-Deve 2017 [92]	- Intervention: University-based (Makerere University) RSS implemented from 2010 to present targeting state and non-state decision-makers, including mid- and top-level officials at Ministries of Health, civil society organisations and legislators - Aim: Timely (within 28 days) development of four-page evidence briefs with clear key messages to support health systems policy and planning decision-making - Evaluation: Qualitative case study entailing interviews with researchers involved in its implementation and policy-makers who used or were conversant with the service, to explore the contextual factors associated with the how and why an RRS may be taken up by users in Uganda in the period 2010 to 2014 - Findings: Buy-in from Ministry of Health, consultation during design and implementation of the service, ongoing sensitisation and reminders, follow-up interviews with users, sustainable funding to run the service, including paying and training full time staff, and RSS research staff maintaining a balance between an institutionalised system and a personal relationship
Syed 2008 [72]	- Intervention: Development and application of the Future Health Systems (FHS) evidence–policy interface conceptual framework based on document review and iterative discussions among FHS research consortium; framework considers four key factors, including the developmental context, research characteristics, decision-making processes and stakeholder engagement - Aim: Tool for assessing research plans’ potential for strengthening research–policy links in LMICs and opportunities for improvement - Evaluation: Applied to six health system research plans - Results: Identified gaps in research plans, including limited focus on the following: nurturing links with institutions involved in decision-making processes, identification and participation on formal and informal networks, and stakeholder analysis to inform the design of engagement strategies - Recommendation: Wide application and evaluation
Individual level	
Mbuagbaw 2014 [93]	- Intervention: 2-day training workshop targeting Cameroonian health researchers’ (university-based and independent) and policy-makers on pragmatic KT trials including distinguishing pragmatic trials from other types of trials, and key concepts in KT, important steps in clinical trial design - Aim: Improve knowledge - Evaluation: Structured pre–post training test survey administered before start of training and immediately after end of training - Finding: Statistically significant short-term improvement in the participants’ mean score (standard deviation) from 14.7 (3.75) in the pre-test to 18.27 (4.21) in the post-test
Activity level	
Lavis 2009 [96]	- Intervention: SUPPORT Tools for evidence-informed health Policymaking (STP) #13: Preparing and using policy briefs to support evidence-informed policymaking - Aim: Guiding questions for preparing policy briefs for use in decision-making processes - Evaluation: not evaluated
Lavis 2009 [95]	- Intervention: SUPPORT Tools for evidence-informed health Policymaking (STP) #14: Organising and using policy dialogues to support evidence-informed policymaking - Aim: Guiding questions for organising policy dialogue - Evaluation: not evaluated
Young 2018 [69]	- Intervention: The Policy BUDDIES Project in Western Cape Province, South Africa, implemented in 2014 for 6 months. Paired/matched provincial policy-makers one-to-one with local researchers skilled in KT skilled and knowledgeable about health policy and systems issues - Aim: To build relationships (termed buddying) between researchers and policy-makers to increase the use of evidence in provincial health policy decisions - Evaluation: External mixed methods evaluation using interviews with policy-makers participating in the programme, focus group discussions with researcher buddies and document reviews - Results: Various uses of evidence observed; evidence presented by researchers not always align with policy-maker evidence needs; researchers’ improved understanding of complexity of policy process, communication of evidence and flexibility; and policy-maker champions and reputation of researchers facilitated programme success

Table summarises the aims, study designs, and results of nine studies reporting on interventions or tools aiming to improve or facilitate the KT capacity and practice of researchers and researcher institutions. It organises the interventions at four levels – systems, institutional, individual and activity. Systems level interventions represent government-led interventions with substantial involvement of academic or research institutions. Institutional level interventions represent those initiated and implemented by academic or research institutions. Individual level interventions aim to improve individual KT knowledge and skills. Activity level interventions are guidelines for implementing specific KT activities such as development of policy briefs, organising policy dialogues and pairing researchers with policy-makers to enhance their interaction.

Of the five studies that presented evaluation results of interventions [69, 91–94], three studies used a qualitative case study design [90, 91, 94], one used a case study design drawing on both quantitative and qualitative data [92], and one used a before and after study design using a survey questionnaire [93]. Two studies assessed the impact of the intervention on improving the links and use of research evidence in health decision-making [69, 94]. One of the two studies verified claims of research impact with a document review [69], the other did not [94]. One study assessed the feasibility of implementing the intervention [92], one focused on understanding factors that would facilitate or hinder uptake of the intervention [91], and one assessed improvement in training participants’ (including health researchers’) KT knowledge [93]. Four focused on the African context [69, 91–93] and one on the Arab context [94].

Across the studies that evaluated interventions aiming to link researchers and policy-makers and promote dialogue and exchange [69, 91, 92, 94], an enabling policy and political environment, such as support from government leadership/policy-maker champions, emerged as critical to the success of researchers’ KT efforts. Other factors cited as crucial were researchers’ reputation and perceived credibility, including knowledge of the context, and researchers' investing time and effort to nurture relationships with target audiences. One study cited a preference for KT platforms to be placed in the policy-making institution to improve researchers’ understanding of the policy-making process [91]. However, another study illustrated the challenge of this in relation to researchers' independence and the quality of research generated, which was reported as diminished due to the use of this approach [94]. Nevertheless, having a structured mechanism for promoting interactions between researchers and policy-makers that is supported by the policy-makers, was reported to have resulted in some benefits, including improving interactions between researchers and decision-makers, raising researchers' awareness of policy questions, research informing policy decisions and, in some cases, policy changes, and an increase in policy-relevant research.

## Discussion

This review presents an overview of published literature on LMIC researchers’ KT capacity and practice and interventions for enhancing their KT practice. To our knowledge, no similar review exists. In fact, researchers’ KT practice has been described as understudied, yet, there is increased pressure for researchers to illustrate the policy and practice impact of their research [97–99].

This review reveals some efforts to document LMIC researchers’ KT capacity, practice and efforts to enhance LMIC researchers' KT capacity and practice but also a need to strengthen the evidence base. A total of 66 relevant publications were identified from earliest records available in the databases that were searched up to February 2019. More than half (59%) of the studies focused on sub-Saharan Africa as either a primary or one of several target study settings. Most of the publications focused on health research and a sizeable proportion of these were case studies, descriptive cross-sectional surveys based on participant self-reports or commentary articles. Research designs used in primary studies that sought to assess the levels or extent of capacity and practice varied, making it difficult to compare and contrast across studies. For instance, some studies, particularly institutional KT capacity assessments, gathered and reported aggregated data from both researchers and policy-makers, while other studies gathered and reported data just from researchers. Most of the papers reported LMIC researchers’ KT practice. Very few studies assessed or reported on interventions aimed at enhancing LMIC researchers’ KT capacity as well as their effect on KT practice. Most studies reporting LMIC researchers' KT practice were based on analyses of specific policy processes that explored the roles, influencing factors and strategies of various actors as opposed to an in-depth look at LMIC researchers’ efforts, gaps and influencing factors.

This review identified three key issues relevant to understanding LMIC researchers’ KT capacity and practice and identifying interventions that improve their KT practice. These include the need for more high-quality research on LMIC researchers’ KT capacity and practice; the need for multifaceted interventions that address both LMIC researchers’ individual and institutional KT capacity and practice gaps; and the need for better designed studies that evaluate interventions seeking to enhance researchers’ KT capacity and practice.

This review reveals a need to generate and publish high-quality research focusing on in-depth analyses of LMIC researchers’ KT capacity and practice and the influencing factors. In addition, KT capacity and practice assessment tools and methods could benefit from some standardisation to aid comparison across research type (basic versus applied), research topic, institutions and contexts. This would help confirm the findings of this review, which suggest an influencing role of research type, topic, institutions and contexts on researchers’ KT capacity. The need for high quality KT literature is not unique to LMIC contexts having also been expressed in KT literature focusing on HIC contexts [8, 100]. Study designs that extend beyond case studies and descriptive studies, use participant observation and documentary evidence, are theory based, draw on policy analysis literature from political science and provide nuanced interpretations of ‘context’, ‘policy’ and ‘research’ have been recommended to improve the KT evidence base in general [8, 101, 102]. A notable gap in studies that reported KT capacity assessments was their lack of investigation of researchers’ interest in KT given that attitude is considered an important predictor of practice [103]. Therefore, studies exploring LMIC researchers’ KT capacity and practice should also investigate researchers' interest in KT and how it influences KT practice.

Despite the noted gaps in the evidence base, the findings of this review suggest that researchers and research institutions allocate more effort and investments to research roles and functions relative to KT roles and functions. In addition, inadequate competency among researchers to undertake KT and lack of or little support for KT by research institutions were cited as the main barriers to LMIC researchers’ KT practice. Notably, across the studies in this review that assessed KT capacity using a quantitative assessment tool, institutional KT capacity emerged worse than individual KT capacity. Specifically, the existence of training courses focusing on KT, funding, guidelines, incentives, institutional linkages with end-user organisations and staff with KT expertise to support researchers were consistently scored in the low range of the measurement scales used. Inadequate research communication and collaboration or interaction between researchers and research end-users was a common finding across the studies in the review. Furthermore, studies that assessed researchers’ interaction and collaboration with research end-users and/or institutional links with research end-user organisations revealed that some critical target groups, such as media, non-government organisations and industry, are left out. Interaction and collaboration, also referred to as ‘stakeholder engagement’, is being increasingly promoted as an important pathway to achieving research-to-policy-and-practice impact [104]. Stakeholder engagement involves working with diverse groups of stakeholders in the research process, giving them shared decision-making authority, and thus taking into consideration their interests and values into research design, implementation and dissemination [104, 105]. This in turn increases the relevance and credibility of the research produced and the chances of its uptake in policy and practice decisions [104, 105]. Inadequate interaction and collaboration between researchers and research end-users is not a unique challenge to the LMIC context and has also been reported in studies in HIC settings [24, 98, 99, 102, 106–110]. The review identified research communication, and researchers' interaction and collaboration with research end-users as the most critical factors that influence researchers’ KT practice. At institutional level, funding for KT and institutional support and incentives promoting KT emerged as the most critical factors that influence researchers’ KT practice.

The most cited strategies for enhancing LMIC researchers’ KT practice in this review align well to the identified LMIC researchers’ KT capacity and practice gaps and the barriers and facilitators of LMIC researchers' KT practice. They include allocating/increasing access to funding for KT, researchers’ skills development, and establishment of institutional links and enhanced interaction between researchers and research end-users and their organisations. Establishing or strengthening institutional incentives to encourage KT did not emerge as one of the top suggested interventions but is nevertheless important to address, as expressed elsewhere [98]. Notably, a few publications recommended strategic partnerships between LMIC and international research institutions as an approach for transferring knowledge and skills and sharing resources in areas where capacity gaps exist. Gaps in tertiary education systems in LMICs are widely documented and include insufficient numbers of qualified academic faculty and budgetary constraints [111]. This limits the capacity of LMIC academic institutions to provide high quality graduate education. The benefits of partnerships between LMIC and international research institutions (North–South partnerships) has been cited elsewhere, although with the caveat that, for these collaborations to work, they need to be equitable [26, 112]. Others have argued for the model to be adapted so that the leadership of such partnerships is the responsibility of the Southern partner [27]. Indeed, partnership models that are being led by Southern partners are currently being tested in research capacity strengthening programmes such as the Wellcome Trust-funded Developing Excellence in Leadership, Training and Science (DELTAS​)​ [128].

Whilst this review identified few published evaluations of KT capacity strengthening interventions for LMIC researchers, we acknowledge previous or current capacity strengthening efforts being implemented across LMICs although not published in peer-reviewed journals. These interventions have largely focused on improving researchers' individual KT capacity [98, 102, 113–115], including training on KT theory and its application, barriers and facilitators; KT strategies and plans; research communication skills; systematic review training; and skills for developing and sustaining relations with policy-makers and the media [98, 102, 113–116]. Some have focused on strengthening collaboration and supporting the establishment and operation of KT support networks [8, 117, 118]. Examples include EVIP-Net and the Consortium for Health Policy and Systems Analysis in Africa [117, 118]. A few examples of efforts to improve the recognition of KT in tenure and promotion processes in academic institutions exist but largely in HIC contexts [98, 99] and the extent to which these policies are promoted, recognised, applied and evaluated is unclear [98]. Currently, there are no examples of multipronged interventions that aim to concurrently enhance LMIC researchers' individual and institutional KT capacity. Yet, this review reveals the need for such multifaceted interventions. For example, one study in this review illustrated that structural changes promoting KT practice by researchers and policy-makers in the absence of policies, legislature and guidelines that mandate and guide institutional behavioural change hindered improvement in KT practice [94]. There is increasing recognition among KT practitioners of the importance of multipronged interventions to achieve sustainable improvement in KT practice [98].

Finally, the review revealed very few published evaluations of KT interventions for improving researchers’ KT practice, which, given the range of researcher-focused KT capacity strengthening initiatives that have been implemented (as described above), reflects a missed opportunity for learning about the effectiveness of KT interventions. We found only nine papers on KT interventions and tools for improving researchers’ KT capacity and practice and, of these, only five reported evaluations of interventions [69, 91–94]. Furthermore, the studies employed heterogenous study designs and methods and had varied objectives and focused on a range of contexts. Despite the heterogenous nature of the evidence base, the review drew some common findings from the five evaluation studies, all of which focused on interventions aimed at linking researchers and policy-makers. These interventions reported a range of benefits, including instrumental, symbolic and conceptual changes in policy decisions. The importance of a supportive leadership in government/policy-maker champions, researchers’ reputation and credibility, and researchers putting in time and effort to build relationships and trust were identified as facilitative. The studies found differences in preferences on the ideal host of a KT platform with one study advocating for a government agency hosting such a platform and another advocating for a non-state institution playing that role.

These findings are useful but are based on a few studies that have mainly used case study and descriptive designs. As a result, these findings fall short of providing strong evidence on the effectiveness of various intervention models on LMIC researchers’ KT practice. The findings point to the need for more evaluation studies to better understand for whom and under what circumstances KT interventions work. KT intervention evaluations are consistently identified as either underrepresented across the global KT evidence base or largely of inadequate quality because they are poorly designed [21, 98, 116, 119–124]. KT practitioners and scholars are advised to employ realist approaches, pragmatic trials, impact evaluations, implementation research and participatory action research, which are more suitable for the evaluation of social and contextually sensitive interventions like those associated with KT [8, 21, 125, 126]. In addition, more attention to the variety of impacts and effects resulting from research has also been recommended [101].

The review has some limitations. Firstly, it focused on published literature, authored in English, thereby potentially excluding relevant evidence reported in the grey literature and/or in other languages. Secondly, while substantial efforts were made to select the most relevant search terms, some key terms may have been missed, leading to the exclusion of relevant evidence. Finally, LMICs are not all the same; therefore, the findings may not be broadly applicable across LMIC contexts, especially given the predominance of sub-Saharan Africa-based studies. Thus, the review findings should be interpreted with this in mind.

## Conclusions

The available evidence suggests that LMIC researchers rarely practice KT, mainly because they face capacity constraints and barriers at individual and institutional levels. Increased access to funding for KT, researchers’ KT skills development, including partnerships with international research institutions, improved links and interaction between researchers and research end-users and their institutions, and institutional incentives promoting KT practice are recommended. More in-depth, high quality research on researchers’ KT capacity, practice and effective KT capacity strengthening interventions is needed, including some standardisation of methods and assessment tools. Study designs that extend beyond case studies and descriptive studies, that are theory based, draw on policy analysis methods, provide nuanced interpretations of ‘context’, ‘policy’ and ‘research’, and pay attention to a range of impacts and effects, are recommended. Furthermore, realist approaches, pragmatic trials, impact evaluations, implementation research and participatory action research are recommended for evaluating interventions. Notably, the findings in this review are largely consistent with what has been reported in HIC settings.

## Supplementary information

**Additional file 1.** List of included primary studies. Presents the list of included primary research studies in this review. It presents information on the study design and methods including sampling, and description of the study population and setting.

## Data Availability

All data generated or analysed during this study are included in this published article.

## Abbreviations

HIC: High-income country
KT: Knowledge translation
LMIC: Low- and middle-income country
MMAT: Mixed Methods Appraisal Tool
NGOs: Non-government organisations
