# Supplementary material for: Review of published evidence on knowledge translation capacity, practice and support among researchers and research institutions in low- and middle-income countries
Source: Health Res Policy Syst. 2020 Feb 10;18:16. doi: 10.1186/s12961-019-0524-0 (PMC7011245; doi:10.1186/s12961-019-0524-0)
Supplement: Supplementary file 1 — Additional file 1. List of included primary studies. Presents the list of included primary research studies in this review. It presents information on the study design and methods including sampling, and description of the study population and setting. [file 12961_2019_524_MOESM1_ESM.docx]

**Additional file 1. List of included primary studies**

List of included primary research studies in the review of published evidence on knowledge translation capacity, practice and support among researchers and research institutions in low- and middle- income countries

| **#** | **Author, year, study focus** | **Study design and methods** | **Study population**  **(Participants, sample size, country, region)** |
| --- | --- | --- | --- |
| 1 | El Jardali 2018  Explore KT activities with emphasis on institutional planning for research, national planning of health research and knowledge management, translation and dissemination | Study design:   - Cross-sectional survey   Sampling   - Purposive   Items/issues explored or measured   - Four items scored using a five-point scale (1=low, 5=high): institutional characteristics; institutional planning for research; national planning of health re­search; and knowledge management, translation and dissemination   Data analysis   - Descriptive analysis by country income level, facility type and sector using SPSS - Thematic analysis of data from open-ended questions - Disaggregated findings presented only where differences in trends observed   Study period:   - August 2015 to July 2016 | - 223 health research institutions - 22 Eastern Mediterranean Region Member States (5 high-income, 12 middle-income and 5 low-income countries) - 38.8% response rate |
| 2 | Sriram 2018  Explore the policy-making process for establishing a health sector knowledge platform in India | Intervention:   - Health sector knowledge platform   Study design:   - Qualitative case study - In-depth interviews and document review   Sampling   - Purposive   Items/issues explored or measured   - enabling contextual factors, the interests and actions of stakeholders, and the varying institutional arrangements explored in the development of the NKP   Data analysis   - Thematic Analysis   Study period:   - October 2015 to January 2016 | - 11 researchers (6 involved in the process & 5 researchers with no prior or current involvement) - 5 policymakers - 42 Meeting minutes, communications & published material on the internet - India |
| 3 | Young 2018  Evaluate intervention for building relationships between researchers and policymakers | Study design:   - External mixed-methods evaluation - document review, structured reflections, in-depth, semistructured interviews & focus group discussion   Sampling   - Purposive   Items/issues explored or measured   - Implementation process, barriers & successes; strategies used and the short-term impact   Data analysis   - Framework thematic analysis   Study period:   - Implemented in 2014 for 6 months | - 5 Researchers & 7 Policymakers - Policy documents - Cape Town, South Africa |
| 4 | Conalogue 2017  Identify future global health research priorities | Study design:   - Cross-sectional survey - Health research expert panel - Internal DfID health adviser consultation, and - Delphi study with expert informants   Sampling   - Purposive/Convenient   Items/issues explored or measured   - Views on (1) the long-term future global health research priorities; (2) areas likely to be less important over time; (3) how to improve research uptake in low-income countries; and (4) how to build research capacity in low-income countries   Data analysis   - Thematic analysis   Study period:   - May to June 2015 | - 421 researchers from academia (43%), non-governmental organisations (23%), international organisations (13%), national government (9%) and industry (3%), high-income countries (60%) & low- or middle-income countries (40%) |
| 5 | Lashari 2017  Assessment of KT practices of  PhD faculty members of universities offering degrees in field of Environment in Sindh Pakistan | Study design:   - Cross-sectional survey - 96% response rate   Sampling   - Purposive   Items/issues explored or measured   - 7 clusters of KT activities including publications, networking, mobility of researchers, joint research, intellectual property and co-operations including spin-off companies and sharing of equipment & 28 sub-factors   Data analysis   - Descriptive analysis using SPSS   Study period:   - July 2015 to December 2015 | - 8 universities offering degrees in field of environment including environmental sciences (3) & environmental engineering (5). - 23 PhD faculty members (12 working in the universities offering degrees in environmental engineering and 12 in environmental sciences) - Sindh, Pakistan |
| 6 | Mijumbi-Deve 2017  Explore the contextual factors associated with the how and why a Rapid Response Service may be taken up by users in Uganda | Intervention:   - RRS at the College of Health Sciences in Makerere University established in March 2010   Study design:   - Case study employing process evaluation methods - in-depth interviews   Sampling   - Purposive   Items/issues explored or measured   - Views on the components that make up the RRS and contribute to the completion of its process including structures and activities   Data analysis   - Thematic analysis   Study period:   - March 2010 to May 2014 | - 11 Researchers (7 affiliated with university) and KT specialists & 10 policymakers (6 based at the ministry of health) involved and/or conversant with program operations - Uganda |
| 7 | Shroff 2017  Analyse institutional capacity for the generation of health policy and systems research (HPSR) and the use of evidence (including HPSR)  more broadly in LMICs | Study design:   - Literature review - Two survey instruments   Sampling   - Purposive   Items/issues explored or measured   - Two thematic areas: 1) capacity of research institutions to generate HPSR; & 2) incorporation of research evidence into decision-making for health, including the capacity of decision-makers to use research evidence   Data analysis   - Descriptive analysis   Study period:   - July to December 2014 | - 101 institutions engaged in HPSR within the the Alliance & represented at the Second Global Symposium on Health Systems Research - Based in 56 countries (25% represented sub-Saharan Africa & 63% low- & middle- income countries) |
| 8 | Sween-Cadieux 2017  Evaluate the implementation of the dissemination  workshop, the effects observed following it and  the processes that facilitated, or did not facilitate, the use of the research results | Intervention:   - 2-day dissemination workshop in November 2013 organised by researchers - Attended by 31 people   Study design:   - Mixed convergent qualitative–quantitative design - Survey & semi-structured interviews - Group discussions of 5 of 10 Policy Briefs (50%) several days after training - 81% response rate   Sampling   - Survey: Census - Interviews: Purposive, contrasted sampling technique   Items/issues explored or measured   - Survey   - Four components: 1) participants’ expectations; 2) their assessment of the workshop’s objectives, content and organisation; 3) their opinions regarding the utility of the knowledge acquired and their intention to use it to improve their practices; and 4) their suggestions for improving future workshops   - Statements scored on a 7-point Likert scale from 1 (strongly disagree) to 7 (strongly agree). - semi-structured interviews, contrasted sampling technique   - activity and the PBs, the knowledge they had acquired, their intention to use that knowledge and how the workshop might be improved   Data analysis   - Descriptive analysis of data from the questionnaires - Thematic analysis of data from interviews and group discussions   Study period:   - November 2013 | - Survey: 25 workshop participants - Interviews: 7 participants (involved in decision-making and public administration (n = 2), program management (n = 1), NGOs (n = 2), health professions (n = 1), CSO (n = 1) - Groups discussion: 40 advisors and program heads in several ministries, physicians and researchers - Burkina Faso |
| 9 | Kok 2016  assess how 30 research projects evolved and the results were translated into action | Study design:   - Contribution Mapping - Interviews and document review   Sampling   - purposive   Items/issues explored or measured   - Three phases examined: 1) formulation phase; 2) knowledge production phase; and 3) the knowledge. dissemination and utilization. For each phase, the main actors, activities and interactions were mapped.   Data analysis   - Framework thematic analysis, within-case & cross case analysis   Study period:   - March 2005 to 2008 | - 113 Researchers (n=54) and potential key users from Ministry of Health, the Ghana Health Service or other health-related organizations (n=59) associated with 30 research projects that are part of Ghanaian Dutch Health Research for Development Program funded between 2002 and 2004 - Ghana |
| 10 | Tesfazghi 2016  To dentify potential challenges  and opportunities for accelerating access to new  vector control tools in Burkina Faso | Study design:   - Desk review to identify key actors (institutions and individuals) involved in national vector control for interview, to refine the research question and semi-structured interview guide, and to supplement findings from these - Semi-structured interviews - Informal discussion with representative of private sector   Sampling   - Purposive   Items/issues explored or measured   - Seven themes: actors, power, context, content and process, availability & affordability   Data analysis   - Framework thematic analysis   Study period:   - April 2014 | - 13 people representing 13 institutions including 2 researchers, 4 staff of the MoH, 3 NGOs, 2UN technical agencies, 2 donors & 1 private sector - Ouagadougou, Burkina Faso |
| 11 | Valinejadi 2016  Assess the status of diabetes KT in Iranian diabetes research centers to find out the strengths and weaknesses of principal institutes undertake producing and disseminating diabetes knowledge in Iran as a developing country | Study design:   - Concurrent mixed methods approach - The self‑assessment tool for research institutes (SATORI) - 24 Focus group discussions (FGDs)   Sampling   - Census - 75% response rate   Items/issues explored or measured   - 50 items in four KT domains scored using five-point Likert scale (1=low, 5=high): “The question of research” (12 items), “knowledge production” (9 items), “knowledge transfer” (25 items), and “promoting the use of evidence” (4 items). Every item of this tool evaluated at least one of the aspects affecting KT   Data analysis   - Descriptive analysis of data from survey using Excel software - Thematic analysis based on SATORI‑extracted seven themes   Study period:   - 2015 | - 65 diabetes researchers from 14 diabetes research centers in Iran |
| 12 | Nabyonga-Orem 2015  Explored policy-makers’ attitudes of what evidence is and which types are important for decision-making and in which hierarchy, if any, in Uganda | Study design:   - Exploratory qualitative method - In-depth interviews   Sampling   - Purposive - stakeholders in health policy development and KT in Uganda at national and sub-national (district) levels   Items/issues explored or measured   - Views regarding suitable evidence to guide policy development   Data analysis   - Content analysis   Study period:   - June 2012 to August, 2013 | - 51 people including 4 researchers (2 from public & 2 from private institutions), 18 MoH, 8 Service providers (4 from public & 4 from private sectors), 1 MoF, 1 journalist, 1 parliamentarian, 6 CSOs & 8 donors |
| 14 | Walugembe 2015  Explore activities implemented by researchers to promote research utilization in reproductive health policymaking processes in Bangladesh | Study design:   - Exploratory case study design - Three case studies - Key informant interviews, focus group discussions & database and document reviews   Sampling   - Purposive   Items/issues explored or measured   - Used the World Health Organization/Turning Research into Practice (WHO/TRIP) framework to examine: the research process, stakeholder engagement, communication and dissemination, as well as macro contextual factors   Data analysis   - Thematic framework   Study period:   - September and December 2011 | - 21 key informants, including 13 researchers, two policy makers, and six programme implementers involved with 19 reproductive health studies conducted and completed by International Centre for Diarrhoeal Disease Research Bangladesh researchers between 2001 and 2011 |
| 15 | Ayah 2014  Analysing existing capacities for Health Systems Research (HSR); building consensus  around HSR capacity development strategies for  each SPH; and making an initial and rapid assessment of  HSR priorities in the different countries involved in the  HEALTH Alliance | Study design:   - Context-adapted and modified self-assessment tool & document review - dissemination workshops to discuss and validate the results of their capacity assessments   Sample   - Purposive - Response rate varied from 9% in Jimma University College of Public Health and Medical Science, Ethiopia to 92% in Kinshasa School of Public Health, DRC   Items/issues explored or measured   - Indicators scored using a 5-point Likert scale (1 = strongly disagree, 5 = strongly agree): the presence of a KT strategy, an organizational structure to support KT activities, KT skills, and institutional links with stakeholders and media   Data analysis   - Descriptive analysis using excel   Study period:   - 2011 | - 123 respondents from all seven member institutions of the HEALTH Alliance Africa Hub, a consortium of seven schools of public health in East and Central Africa including Makerere School of Public Health, Uganda, Kinshasa School of Public Health, DRC, Muhimbili School of Public Health, Tanzania, Moi University School of Public Health, Kenya, National University of Rwanda School of Public Health, Rwanda, Jimma University College of Public Health and Medical Science, Ethiopia, University of Nairobi School of Public Health, Kenya |
| 16 | Corluka 2014  Explores researchers’ roles in evidence-informed decision making and proposes a new framework for thinking about how researchers interact with (and can influence) their working environment | Study design   - Qualitative, constructivist epistemological approach - Semi-structured, in-depth interviews   Sampling   - Purposive and snowball sampling   Items/issues explored or measured   - experiences in informing health policies or programmes with their research, working with policymakers in the Argentine public health sector, perceptions of policymakers and the policymaking process in Argentina & facilitators of or barriers to research use in policymaking   Data analysis   - Thematic analysis, grounded theory   Study period:   - May and August 2008 | - 20 health research (biological and social science) in the Federal City of Buenos Aires and the provinces of Salta, Jujuy, Tucuman, Santiago del Estero and Catamarca - University-based, in a combined research and decision-making capacity for provincial Ministries of Health, or within nongovernmental organizations (NGOs), such as think tanks. |
| 17 | Goyet 2014  Evaluation of a ‘knowledge translation’ (KT) intervention to identify the barriers to KT encountered in this LIC setting, and to suggest strategies to facilitate KT in similar settings | Intervention:   - A multidisciplinary working group of national and international clinicians, biologists, health program managers and epidemiologists involved in pneumonia management in Cambodia, dubbed the CALIBAN network formed to provide the MoH with locally relevant and evidence-based knowledge on pneumonia to inform the revision of the pneumonia national guidelines   Study design   - Retrospective evaluation - Review of document review, email correspondence & 1 key informant interview   Sampling   - Purposive   Items/issues explored or measured   - Policy content analysis against CALIBAN key messages - AGREE-II online tool: three appraisers independently score six domains, i.e., scope and purpose, stakeholder involvement, rigor of development, clarity of presentation, applicability, and editorial independence. Scores of appraisers are then summed up and standardized domain scores computed (expressed on a scale of 0–100). - Unified Modeling Language (UML) graphical tools used to frame and analyze the dynamics of activities, the interactions between actors and the documents’ exchanges during the adult guideline updating   Data analysis   - List & group barriers and facilitators to KT   Study period:   - 2013 | - Project protocols, reports, published literature, and meeting minutes from CALIBAN, successive drafts of guidelines - Researchers, the CALIBAN network and the Task force - Representative of NGO who assisted the guidelines update |
| 18 | Maleki 2014  Assess and compare the KT status of selected health research institutes in the Eastern Mediterranean Regions (EMR) countries, and to identify their strengths and weaknesses in the field | Study design   - The ‘Self-Assessment Tool for Research Institutes’ (SATORI) tool - 15 FGD (A minimum of 6 and a maximum of 8 participants (mean number of participants = 7) - 40% response rate   Sampling   - Purposive   Items/issues explored or measured   - 50 items classified into 7 main domains scored using a five-point Likert scale (1=high, 5=high): ‘priority setting’, ‘research quality and timeliness’, ‘researchers’ KT capacities’, ‘facilities and pre-requisites of KT’, ‘processes and regulations supporting KT’, ‘interaction with research users’, and ‘promoting and evaluating the use of knowledge’   Data analysis   - Descriptive analysis of quantitative data - Thematic analysis of qualitative data - Data dissagregated by income status (but not all reported)   Study period:   - 2011 | - 8 medical universities & health research institutes in 8 EMR countries (high-income, low-income & middle-income countries). - 90-120 participants including Deputy and/or director of research affairs, two researchers (at least one professor, one associate professor, one of whom was a lady) and stakeholders from research utilizing organizations, especially the Ministry of Health (MoH). |
| 19 | Mijumbi 2014  The feasibility of a Rapid Response Service (RRS) to meet the urgent needs of policymakers for evidence about health systems | Intervention:   - RRS at the College of Health Sciences in Makerere University established in March 2010   Study design   - Case study - Document review - Key informant interviews   Sample   - Purposive   Items/issues explored or measured   - How service was used, immediate and delayed (after one month) & experiences of the users following receipt of the rapid response evidence briefs   Data analysis   - Descriptive analysis of quantitative data - Thematic analysis of qualitative data   Study period:   - March 2010 to July 2012 | - Questionnaires & other resources used on the service and for the study - Policymakers |
| 20 | Nabyonga-Orem 2014  Examine the uptake of evidence in public health policy making in Uganda | Study design   - cross-sectional qualitative study - In-depth interviews   Sample   - Purposive   Items/issues explored or measured   - Perceptions of the role of evidence in public health policy development, their understanding of KT and their views on the appropriateness of different KT activities that are currently implemented in Uganda   Data analysis   - - content analysis   Study period:   - Not indicated | - 17 participants including 5 from MoH, 4 CSOs, 1 private for profit & 5 donors and 2 researchers - Members of the Health Policy Advisory Committee (HPAC), the policy advisory body for the health sector |
| 21 | Simba 2014 | Study design   - Self-assessments, key informant interviews (KIIs) of internal & external stakeholders, and a review of documents - Plenary meeting to validate the findings   Sample   - Purposive - Response rate varied from 9% in Jimma University College of Public Health and Medical Science, Ethiopia to 92% in Kinshasa School of Public Health, DRC   Items/issues explored or measured   - Self-assessment tool: adequacy of academic members of the SPH to engage in HSR; availability and access to research funding for HSR; the external and internal organizational environment under which research is conducted & individual researchers’ motivation to conduct research - Interviews: Contextual factors influencing the conduct of HSR within the country including, existing policies, available human and financial resources, and staff motivation for HSR   Data analysis   - Descriptive analysis of quantitative data - Thematic analysis of qualitative data   Study period:   - 2011 | - 123 researchers from seven member institutions of the HEALTH Alliance Africa Hub (See Ayah 2014) - 73 Deans of the schools of public health, Ministry of Health, donors and non-governmental officials |
| 22 | Gholami 2013  Assess the status of knowledge translation (KT) in Iranian medical  science universities in order to assess the strengths and weaknesses | Study design   - Cross-sectional study - The Self-Assessment Tool for Research Institutes (SATORI) - 16 FGDs   Sampling   - Stratified random sampling to select institutions - Purposive, maximum variance   Items/issues explored or measured   - 50 items categorised into 7 themes scored using a five-point Likert scale: “priority setting, researchers KT capacities, interaction with research users, the facilities and prerequisites of KT, processes and regulations supporting KT, and promoting and evaluating the use of evidence”   Data analysis   - Descriptive analysis of quantitative data - Thematic analysis of qualitative data   Study period:   - Not indicated | - 9 Iranian medical universities, affiliated to the MoHME - University’s vice chancellor or the director of research, the members of the research committee, and researchers (at least two faculty members who had published at least three articles relating to applied research) - Other stakeholders (one from the healthcare system and one from other organizations such as pharmaceutical companies, the medical equipment industry and/or a public sector domain other than health) |
| 23 | Nabyonga-Orem 2013  Examine the roles and links among various stakeholders in KT as related to public health policy | Study design   - Qualitative study - document review & indepth interviews   Sampling   - Purposive   Items/issues explored or measured   - Perceptions about the roles of various stakeholders in KT, the challenges faced by these stakeholders, and the availability of platforms for stakeholder engagement   Data analysis   - Deductive content analysis   Study period:   - November 2010 to January 2011 | - 15 members of the Health Policy Advisory Committee (HPAC) including government officials at the central level (n = 4), service providers at the district level (n = 1), and representatives of CSOs including coordinators (n = 2) and service providers (n = 2) - Representatives from private for-profit (n = 1) organizations, multilateral donors (n = 3), bilateral donors (n = 2), researchers (n = 2), journalists/media (n = 2), and parliamentarians (n = 2) were interviewed. |
| 24 | Campbell 2012  Case study of a “dissemination as intervention” methodology to  report back research findings to study communities in ways that seek to facilitate community responses to pressing social problems | Study design   - Case study - Structure discussions - Post-workshop evaluation reflections   Sampling   - Purposive   Items/issues explored or measured   - Effect of intervention on awareness of AIDS, critical understandings of the community’s lacklustre response, ownership of the problem, a recognition of local resources for responding more effectively & awareness of potential support partners outside of the community   Data analysis   - Thematic content analysis   Study period:   - Not indicated | - Intervention targeted to 9 local groups involving 121 participants (78 women and 43 men) |
| 25 | El-Jardali 2012  Explore how researchers view and experience the role of health systems research in health policymaking in the EMR, including the factors that influence health policymaking and the factors that increase researchers’ engagement in KTE activities | Study design   - Cross-sectional survey   Sampling   - Purposive – Corresponding authors who had published relevant articles between the years 2000 and 2008 in local or international journals indexed on Medline or EMBASE - 56% response rate   Items/issues explored or measured   - Four main quantitative scales, and seven open-ended questions scored using a five-point scale (1=high, 5=high) assessing: researchers’ KT activities, skills and necessary KTE training and have undertaken KTE activities, investments/ resources available to researchers to support their KTE activities, policymakers’ usage of evidence in addition to the factors the influence the use of evidence in policymaking, health policymaking context in the region, needs to ensure that research is transferred to health policymakers and stakeholders   Data analysis   - Descriptive analysis of quantitative data & Linear Regression Models - Thematic analysis of qualitative data   Study period:   - Not indicated | - 133 health systems and policy researchers |
| 26 | El-Jardali 2012_2  Assess the climate for the use of evidence in policy & explore views and practices on the current processes and weaknesses of  health policymaking | Study design   - Multi-staged study - Questionnaires - Discussion of case study scenarios on health policymaking   Sampling   - Purposive - 64.3 % response rate   Items/issues explored or measured   - Three quantitative scales including seven items that assessed the availability of health research evidence about high-priority policy issues, five items that assessed the strength of relationships among policymakers & researchers & four items that assessed policymakers' capacity to support the use of health research evidence in health systems policymaking scored using using a seven-point Likert scale (never, very rarely, rarely, occasionally, frequently, very frequently, always)   Data analysis   - Descriptive analysis of quantitative data & Linear Regression Models - Thematic analysis of qualitative data   Study period:   - December 2010 | - 27 participants including 15 Senior policymakers, 4 stakeholders and 8 researchers from Algeria, Bahrain, Egypt, Iran, Jordan, Lebanon, Oman, Sudan, Syria, Tunisia, and Yemen |
| 27 | Nixon 2012  Describe use of  KTE principles to inform the dissemination of survey results to adolescent study participants in  an HIV research project based in South Africa | Intervention:   - Two-way communication approach   Study design   - Researchers’ critical reflections   Sampling:   - Purposive   Items/issues explored or measured   - Strengths, weaknesses and surprises in the dissemination processes   Data analysis   - Written reflection report after dissemination session in each school   Study period:   - October 2008 | - Researchers’ (intervention implementers) critical reflections |
| 28 | Uneke 2012  Describe workshop was used as a forum for the promotion of evidence-informed policymaking because of the many strategic benefits of a workshop | Intervention:   - One-day evidence-to-policy forum (workshop) was held in Abakaliki, the capital of the Ebonyi State in south-eastern Nigeria   Study design:   - Pre & post workshop surveys & FGDs - 79% response rate   Sampling:   - Purposive   Items/issues explored or measured   - health policymaking process and capacity to use evidence & level of involvement of researchers in the research to policy process scored using four-point Likert scale (1=low, 4=high)   Data analysis   - Descriptive analysis   Study period:   - December 2009 | - Six senior academic researchers (Professors and Associate Professors) from the Ebonyi State University, Abakaliki, Nigeria - 81 policymakers & representatives of NGOs, health worker association & police force |
| 29 | Crichton 2011  Collaborative analysis with researchers and communicators in four research programme consortia  (RPC) working on sexual and reproductive health  (SRH) | Study design:   - Qualitative study, participant-observer approach - In-depth interviews, case studies and an interactive workshop   Sampling:   - Purposive & convenience   Items/issues explored or measured   - The role of research in policy, and experiences with policy engagement drawing on the RAPID analytical framework in ordering and presenting our results adapted by adding sphere on the characteristics and actions of researchers and their institutions   Data analysis   - Thematic analysis   Study period:   - August 2008 to May 2009 | 22 participants including 15 researchers and 7 communications specialists in the four RPCs (9 from northern and 13 from southern partners) |
| 30 | Delaney-Moretlwe 2011  Interactions between researchers and society, and the role of researchers as agents of social change | Study design:   - Case study - Accumulated experience, reflections and discussions of the authors over the life of these four trials conducted in Johannesburg, South Africa from 2004-2008. - Document review - Interactive workshop   Items/issues explored or measured   - lessons learnt from communicating the results of four trials evaluating treatment for herpes simplex virus type 2 (HSV-2) as a new strategy for HIV prevention   Sampling   - Purposive   Data analysis   - Not indicated   Study period:   - May 2009 | - Study investigators - Research dissemination plans drafted prior to trial completion, as well as reports written by staff which recorded various aspects of the results communication process, including reactions to the process by stakeholders |
| 31 | Gholami 2011  Designing & pilot a self-assessment tool for knowledge  translation activities in research-producing institutes, Tehran University of Medical Sciences research centres and faculties, Iran | Study design:   - The self‑assessment tool for research institutes (SATORI) - FGDs   Sampling   - Purposive - 100% response rate   Items/issues explored or measured   - 50 statements in four main domains including: the question of research, knowledge production, knowledge transfer & promoting the use of evidence using a five-point Likert scale (1=low, 5=high)   Data analysis   - Descriptive analysis   Study period:  Not indicated | - 12 research centres and 8 departments (One department from each faculty; in the medical faculty, a clinical & a basic science department) at the Tehran University of Medical Sciences, Iran - 120 participants (6 members of the research council and the researchers from different centres and departments) |
| 32 | Ssengooba 2011  Understand the process of translating research into  policy in order to improve health outcomes related to national health priorities in Uganda & explore strategies for academic institutions  like Makerere University College of Health Sciences (MakCHS) to influence the translation of research into policy and practice | Study design   - Case study - In-depth interviews   Sampling   - Purposive & snowball   Items/issues explored or measured   - lessons on how research influences policy developments in Uganda, informed by a number of frameworks linking the research-policy interface   Data analysis   - Mixture of Manifest and Latent content analysis techniques   Study period:   - 2009 | 30 in-depth interviews including 8 researchers,  12 policy makers, and 10 media journalists involved in decision making around or health reporting on the PMTCT and or SMC process at any time since the year 2000 in Uganda |
| 33 | Oronje 2011  APHRC’s experience in working  with the media to promote reporting on health research  in general, and its own research, in particular over the  period 2004 and 2009 | Study design:   - Case study - Personal experiences and reflections of the authors (who played a central role in developing and implementing the Center’s communication and policy engagement strategies) - Survey of science journalists in Kenya conducted in 2007 by the Media for Environment, Science, Health and Agriculture in Kenya (MESHA) on behalf of APHRC - Literature review   Sampling   - Purposive   Items/issues explored or measured   - approaches used and highlight what worked well, the challenges we faced, and overall lessons learnt in order to provide a learning platform for institutions seeking to adopt similar strategies   Data analysis   - Not indicated   Study period:   - 2004 to 2009 | - Study authors - 18 Kenyan science journalists |
| 34 | Tulloch 2011  Explore SRH research to  policy and practice interactions in Sub-Saharan Africa | Study design:   - Case study - Reflections & interactive workshop - Used Sumner et al’s synthesis approach and Nutley et al’s research use continuum to identify lessons and the dominant types of research use   Sampling   - Purposive   Items/issues explored or measured   - Two criteria assessed: role of developing research to policy networks which act on new research evidence; & role of research, advocacy and engagement   Data analysis   - Not indicated   Study period:   - Not indicated | - Study authors & other researchers, communication specialists and donors working with DFID-funded SRH and HIV Research Programme Consortia |
| 35 | Whiteside 2011  Track & evaluate the Reviewing Emergencies report | Study design:   - Qualitative Case study - Literature review of relevant policy documents, articles, op-eds and minutes of key meetings - Questionnaire on influence, potential influence and barriers to influence across sectors ranked using scale from ‘no influence’ to a ‘very large influence’ (including a ‘don’t know’ option) & asked for examples or description of influence - Interviews with people who had significant involvement in the creation dissemination of the report.   Sampling   - Purposive - 40% response rate for survey   Items/issues explored or measured   - Document the creation and dissemination of the report; Identify and explain its impact; Identify any barriers and/or limitations to its impact; & Draw lessons for maximising the impact of future   Data analysis   - Descriptive analysis of quantitative data - Thematic analysis of qualitative data   Study period:   - mid 2008 | - Survey completed by 20 individuals in the five sectors (donors; government; civil society and non-governmental organisations; academia and the media) - 5 interviews |
| 36 | Lavis 2010  Describe the findings from a survey of health care providers  in these countries who were practising in one of these clinical  areas about their awareness of, access to and use of research-based evidence in these clinical areas and the influence of such  evidence on their professional practice | Study design:   - Self-administered questionnaire   Sampling   - Purposive/census - 67.6% response rate   Items/issues explored or measured   - Two main sets of questions scored using five-point Likert scale (1=low, 5=high): one set addressed researchers’ activities in bridging the gaps between research, policy and practice grouped into three broad domains (“producer-push”, efforts to facilitate “user pull” & exchange efforts); The other addressed potential system-level, organizational and individual correlates of researchers’ engagement in these bridging activities   Data analysis   - Descriptive analysis and logistic regression models   Study period:   - April 2004 to April 2005 | 368 researchers in 10 LMICs (China, Ghana, India, Iran, Kazakhstan, Laos, Mexico, Pakistan, Senegal and Tanzania) who conducted research in  one of four clinical areas (malaria prevention, contraception, childhood diarrhoea & tuberculosis (TB) treatment |
| 37 | Garnett 2009  Illustrate that participatory research can be both empowering and transformative regularly leading  to outcomes that extend beyond the life of individual research  projects | Study design:   - Case study   Sampling   - Purposive   Items/issues explored or measured   - Knowledge transfer evidence or benefits   Data analysis   - Not indicated   Study period:   - Not indicated | - 6 case studies in Natural Resource Management (NRM); 2 from Africa and 4 from remote northern and central Australia |
| 38 | Woelk 2009  Understand the process  of knowledge translation in LMICs by describing the factors affecting the use of research findings, particularly findings from RCTs, in national policy development & how actors in the policy process understand the notion of 'evidence' for decision-making | Study design:   - Qualitative case-study - key informant interviews, reviewed documents and developed timelines of key events   Sampling   - Purposive and snowball   Items/issues explored or measured  - Evidence uptake in the policy making process at national  level  Data analysis   - Thematic analysis, cross-country and cross-case study analysis   Study period:   - April 2004 and March 2005 | - 39 participants involved in MgSO4 policy process including 1 regionally-based researcher, 11 government health officials, 12 pharmaceutical policymakers & 3 representatives of pharmaceutical companies, 15 clinician researchers - 47 participants involved in Malaria policy process including 3 regionally-based researchers, 16 government health officials, 5 NGOs, 13 clinician/researchers & 10 international/ bilateral agencies - Regionally-based researchers based in three countries (Mozambique, South Africa and Zimbabwe) in PraCTiHC (Pragmatic Randomized Controlled Trials in Health Care), a project funded by the European Commission |
| 39 | Yousefi 2009  Assess the views of researchers, health policy and decision makers, and research policy makers and support staff on how the development and usage of evidence from systematic  reviews can be promoted in a country with limited resources | Study design:   - Questionnaire   Sampling   - Purposive - 87% response rate   Items/issues explored or measured   - Rank a list of 20 items that potentially influenced attitude and behaviour towards producing and using systematic reviews & strategies for addressing them   Data analysis   - Descriptive analysis & Multidimensional scaling (MDS) method - Content analysis for qualitative data   Study period:   - Not indicated | - 131 participants including clinical and health care researchers, research affairs of Iranian medical universities and their information and research managers, health managers and investigators from the ministry of health |
| 40 | Daniels 2008  Explores the actual and perceived utilisation of research information,  in particular findings from RCTs, in policy making and clinical guideline development for the treatment of  eclampsia and pre-eclampsia in South Africa over the period of 1970 to 2005. | Study design   - Qualitative case-study - Literature review, policy document review, timeline of key events and interviews   Sampling   - Purposive   Items/issues explored or measured   - Policy document review: Establishing the extent to which research information had been implicitly and explicitly used - Interviews: the respondent's background; their knowledge of national policies; their knowledge of and involvement in the policy development process; their understanding of the various influences on the policy process and content including stakeholder involvement, prevailing values and research information   Data analysis   - Thematic content analysis   Study period:   - 1970 to 2005 | 15 local researchers and policy makers |
| 41 | Nedjat 2008  Determine the frequency of various knowledge transfer activities applied by researchers at TUMS & determine factors leading to the type of strategy ('active' or 'passive'). | Study design   - Document review using a checklist - Researcher's questionnaire (self-administered)   Sampling   - Purposive/census - 95.6% response rate for documents reviewed - 74% response rate for survey   Items/issues explored or measured   - List of KT passive and active activities to be selected by respondents & open-ended question for the activities that were not listed in the above-mentioned questions. A score of zero was given if the activity was not carried out; a score of one if it was performed once, and a score of two if it was done more than once. Estimates of percentage of time, or 'percent effort' allocated to each activity, including research, education, clinical service delivery, executive responsibilities, and others & estimate their percent effort in a way that the sum would be equal to 100   Data analysis   - Descriptive analysis & multi-variable linear regression   Study period:   - 2004 to 2006 | - 301 TUMS research projects that received grants from inside and outside the university in 2004 and were completed by the time this study was performed (the second half of 2006) were studied |
| 42 | Block 2003  Analyse institutional structure and characteristics, engagement  with stakeholders, institutional capacity, level of attainment  of critical mass and the process of knowledge production by institutions in low- and middle- income countries. | Study design   - Survey for assessment of capacity to produce, demand or support HPSR, including countries in both North and South   Sampling   - Purposive - 61% response rate   Items/issues explored or measured   - Six strategic and interrelated groups of variables: institutional/country context and characteristics, institutional capacity and engagement with stakeholders, attainment of critical mass of researchers to produce quality, sustainable research, and the process of knowledge production (project portfolio characteristics, including external research project funding).   Data analysis   - Descriptive analysis   Study period:   - 2000 to 2001 | - 108 the Alliance partner institutions producing health policy and systems research in low- and middle- income countries (50 based in low-income, 36 in lower middle- income & 22 in upper middle-income countries) - LICs with most sampled institutions (in order) India, Bangladesh, Pakistan, Indonesia, Kenya, Uganda and Ghana. Those for LMICs are China, Colombia, Philippines, Thailand, Bolivia, Cuba and Sri Lanka; while those for UMICs are Argentina, Brazil, South Africa, Mexico, Korea, Rep., Uruguay and Chile |
| 43 | Trostle 1999 | Study design   - Case study - In-depth interviews, document review of policies & validation meeting - November 1994 to June 1995   Sampling   - Purposive   Items/issues explored or measured   - Analyse content, actors, process & context of each policy process   Data analysis   - Thematic analysis   Study period:   - Mid-1994 | - 67 researchers & policymakers |

HPSR=Health policy and systems research HPSR; NGOs=non-government organisations; CSO = civil society organisations; MoH = Ministry of Health; UN=United; Nations; TUMS = Tehran University of Medical Sciences; LMICs = low- and middle-income countries; LICs = low- income countries; CALIBAN network = Community-Acquired Lung Infections, Bacteria and Antimicrobial Network; the Alliance = Alliance for Health Policy and Systems Research
